# Design and characterization of nanostructured electroactive tetraaniline prepared using alternative oxidizing systems

**DOI:** 10.1039/d6ra06797b

**Published:** 2026-11-02

**Authors:** Apparao Thota

**Affiliations:** a Polymers and Functional Materials Division, CSIR-Indian Institute of Chemical Technology (CSIR-IICT) Hyderabad-500007 India; b Research and Development Division, SNF (India) Pvt. Ltd. Jawaharlal Nehru Pharma City Visakhapatnam – 531 019 India apparaougc80@gmail.com

## Abstract

Emeraldine-base tetraaniline (TAni) was synthesized *via* the oxidative coupling of *N*-phenyl-*p*-phenylenediamine using a series of oxidizing agents, namely, ceric ammonium nitrate (CAN), copper(ii) chloride (CC), ammonium persulfate (APS), benzoyl peroxide (BPO), and iron(iii) chloride (FeCl_3_). This study establishes a systematic comparison of these chemical routes and their influence on the structural, thermal, morphological, electrical, and electrochemical properties of the resulting oligomers. Structural characterization using FT-IR, ^1^H NMR, ESI-MS, CH elemental analysis, and UV-Vis spectroscopy confirmed the successful formation of emeraldine-base TAni with a benzenoid–quinoid framework. TGA, DSC, and FE-SEM analyses revealed a semi-crystalline morphology and good thermal stability under nitrogen. After doping with 1 M HCl, all the tetramers—CAN-TAni, CC-TAni, BPO-TAni, APS-TAni and Fe-TAni_bp_—exhibited higher electrical conductivity than Std TAni (3.0 × 10^−3^ S cm^−1^), which was attributed to their porous nanostructured morphology. Their conductivity followed the order BPO-TAni > CC-TAni > CAN-TAni > Fe-TAni_bp_ > APS-TAni > Std-TAni. Cyclic voltammetry showed three well-defined redox transitions corresponding to the LEB → EB → pernigraniline transformations, closely resembling the electrochemical behavior of polyaniline. These findings demonstrate that controlled oxidative strategies enable the preparation of structurally robust and electrochemically active tetraaniline with tunable conductivity and morphology, offering promising potential for applications in organic electronics and redox-functional materials.

## Abstract 2

Aniline tetramers synthesized using different oxidizing agents exhibited enhanced electrical conductivity, high thermal stability, and reversible redox behavior, highlighting their potential in organic electronics and electrochemical applications.

## Graphical abstract

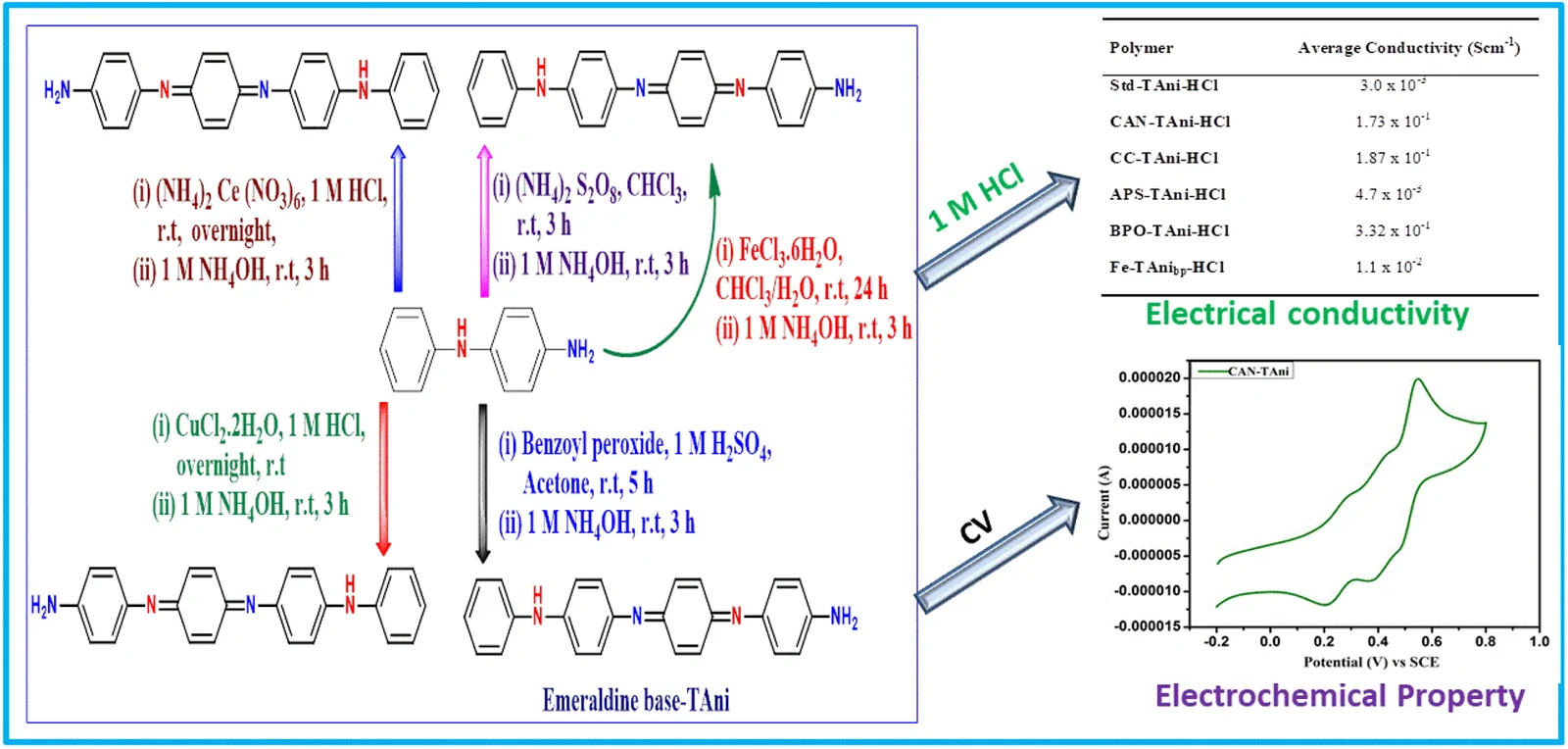

## 1 Introduction

Over the last two decades, intrinsically conducting polymers (ICPs) have emerged as prominent materials for advanced applications^1^ because of their interesting electrical, optical, and chemical properties.^2–6^ These materials have become key components in advanced functional systems due to their structural versatility and tunable performance. These conducting polymers with heteroatoms such as nitrogen (N) and/or sulfur (S) play an important role in various applications. Representative examples include polyaniline (PANI), polypyrrole (PPy), polythiophene (PTh), and poly(phenylene sulfide) (PPS) and their derivatives, all of which exhibit promising electroactive behavior.^7–9^

The importance of precisely controlled molecular architectures and structure–property relationships has been recently highlighted across diverse areas of advanced materials research, including functional nanostructures, heterogeneous catalysis, electrocatalysis, energy-related materials, and biomedical systems.^10–13^ For example, tailored molecular structures and nanostructured interfaces have been shown to influence therapeutic performance, catalytic activity, hydrogenation pathways, ionic transport, and electronic properties.^14–16^ These studies demonstrate that controlling molecular architecture, active sites, interfacial interactions, and nanoscale organization is essential for tuning the functional properties of advanced materials. Such structure–property relationships are particularly relevant to functional polymers, whose molecular structure, conjugation length, oxidation state, and chain organization can strongly influence their electrical, optical, electrochemical, and morphological characteristics.^17–19^

Polyaniline has emerged as one of the most extensively investigated conducting polymers, particularly following the recognition of pioneering contributions to this field with a Nobel Prize in 2000. Polyaniline has been prepared *via* chemical or electrochemical oxidative polymerization of aniline monomers, as demonstrated in the literature.^20–22^ It has attracted considerable research interest because of its tunable oxidation–reduction chemistry, scalability, stability under ambient conditions, good electrical conductivity, and relatively low-cost starting materials, reinforcing its significance in research and industrial applications.^23–27^ Owing to these advantageous properties, polyaniline has found applications in various fields, including electrochemical sensors,^28–30^ electrochromic devices,^28^ electromagnetic interference (EMI) shielding,^31^ supercapacitors,^32,33^ corrosion-protection coatings,^34–36^ and rechargeable batteries.^37^ Nevertheless, challenges associated with its complex molecular structure, limited solubility in common organic solvents, and poor processability hinder its integration into practical devices. In particular, its poor solubility in highly polar solvents such as DMF, DMSO, and NMP presents additional challenges, motivating continued efforts to improve its physicochemical properties.^38,39^

Therefore, to overcome the limitations associated with its processability and structural complexity, significant attention has been focused on low-molecular-weight analogues of polyaniline. Aniline oligomers offer an opportunity to design and produce defined and well-characterized compounds with defined functionality and properties. The oligomers can be synthesized in high purity; they can be used to study the relationship between chain length and physical properties.^40^ Therefore, oligo(anilines) serve as an effective model system for studying the electronic, magnetic, optical, and structural properties of polyaniline.^41–43^

Important oligoanilines, including trimers, tetramers, pentamers, and octamers, have been successfully synthesized and investigated.^44–48^ Depending on their terminal substituents, these oligomers can be classified as phenyl/NH_2_-, phenyl/phenyl-, and NH_2_/NH_2_-end-capped derivatives. **The significance of these oligomers in understanding conjugated polymers is highlighted by studies of phenyl/phenyl-end-capped octaaniline, which have demonstrated physicochemical properties resembling those of polyaniline.^49,50^

Like polyaniline, oligoanilines display three principal oxidation forms: leucoemeraldine, emeraldine and pernigraniline states. Many of their properties are very similar to those of polyaniline. The oligoaniline tetramer possesses the same three redox forms, namely, the fully reduced ‘leucoemeraldine base’ form (LEB), the half-oxidized/reduced ‘emeraldine base’ (EB) form and the fully oxidized ‘pernigraniline base’ form (PB).^51,52^ An electrically conductive state arises from the protonation of the emeraldine base to generate the emeraldine salt form, which can be accessed by simple acid–base redox chemistry (affecting the protonation states) as shown in Scheme 1.

**Scheme 1:**
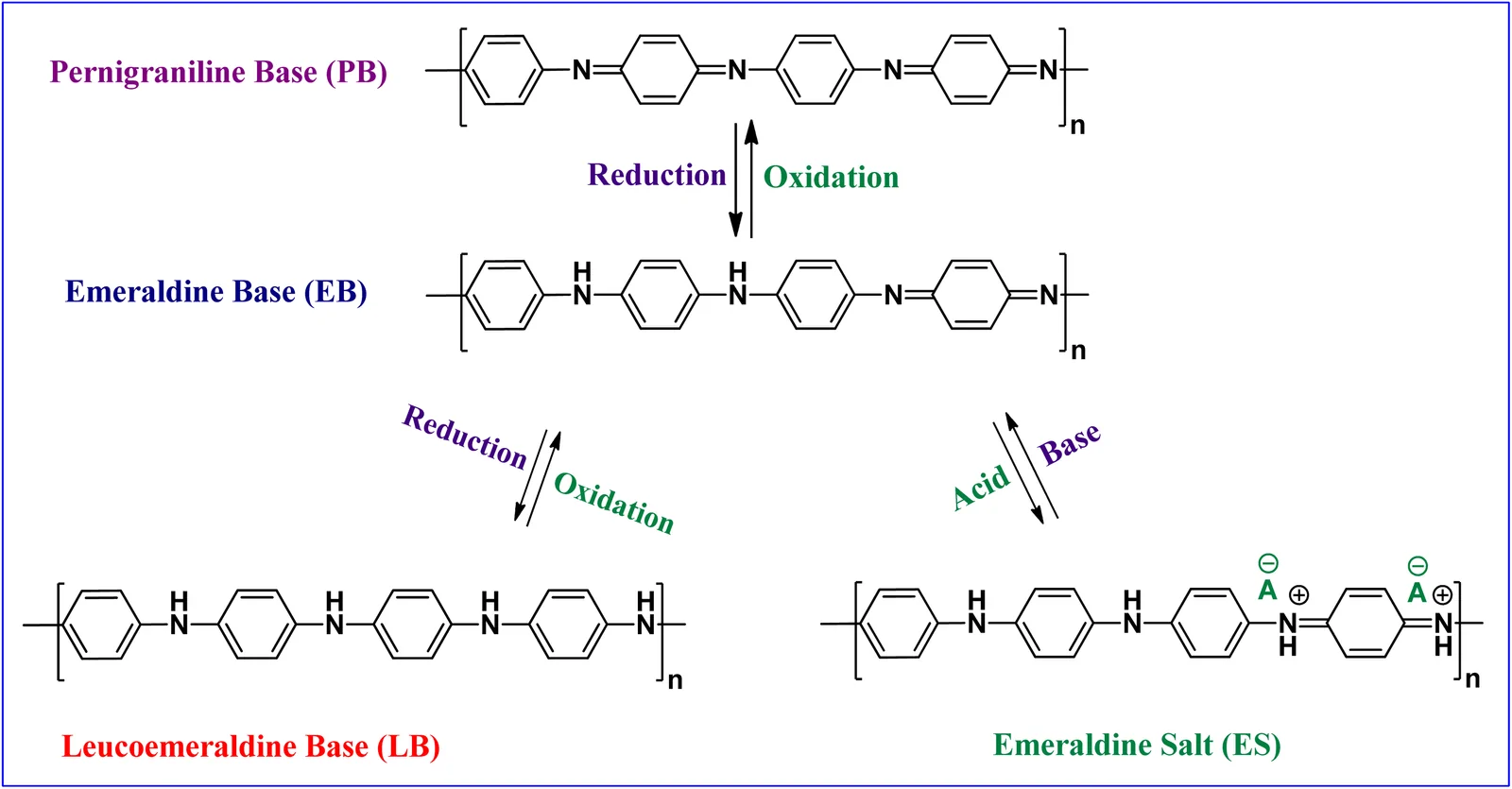
Schematic diagram of PANI/oligoanilines in different oxidation states.

Protonic doping plays a central role in imparting electrical conductivity to oligoanilines. Upon treatment with a strong acid, such as hydrochloric acid, protonation occurs at the nitrogen sites along the backbone, facilitating charge-carrier formation and enhancing electrical conductivity.^53^ These charge carriers, known as polarons and bipolarons, contribute to electronic transport in oligoanilines, as in polyaniline (PANI). The electrical conductivity of tetraaniline is approximately 10^−3^ S cm^−1^, which is lower than that of PANI (approximately 10^3^ S cm^−1^), owing in part to its shorter conjugation length and smaller molecular size. Therefore, tetraaniline is an important molecular model for understanding the emeraldine oxidation state of polyaniline and is considered a well-defined oligoaniline representing this oxidation state.^54^ Emeraldine-state polyaniline contains approximately 50% amine and 50% imine nitrogen sites, with benzenoid and quinoid units in its backbone. Recent studies have focused on synthesizing oligoanilines *via* simple chemical oxidative coupling reactions, often yielding asymmetrically end-capped products with a phenyl group at one end and an amino group at the other.^55–57^ Such strategies aim to produce electroactive materials with improved solubility and processability for device fabrication. Higher aniline oligomers have been synthesized *via* chemical^56,58,59^ and electrochemical methods.^5^ Tetraaniline, an important member of the oligoaniline family, has been synthesized using both chemical oxidative and electrochemical methods. These electrochemical approaches demonstrate the feasibility of controlling oligoaniline formation through electrochemical routes. However, systematic studies comparing different chemical oxidizing systems and correlating the structure and morphology of the resulting tetraaniline with its electrical and electrochemical properties remain relatively limited. The present work focuses on the systematic investigation of alternative chemical oxidizing systems and their influence on the structural, morphological, thermal, electrical, and electrochemical properties of tetraaniline. Although different oxidizing systems can yield tetraaniline with the same fundamental molecular framework, the nature of the oxidant can significantly influence the reaction pathway, oxidation environment, and polymerization kinetics, thereby affecting the physicochemical and functional properties of the resulting material. Therefore, systematic comparison of alternative oxidizing systems is important for understanding their influence on morphology, thermal stability, electrical conductivity, and electrochemical behavior. In the present study, ceric ammonium nitrate (CAN), copper(ii) chloride (CC), ammonium persulfate (APS), benzoyl peroxide (BPO), and iron(iii) chloride (FeCl_3_) were comparatively investigated for the synthesis of tetraaniline to establish the relationship between the oxidizing system and the properties of the resulting material.

## 2 Experimental section

### 2.1 Materials

*N*-Phenyl-1,4-phenylenediamine (DA, 98%), ferric chloride hexahydrate (98%), benzoyl peroxide (BPO, 75%), ammonium peroxodisulfate (APS, 98%) and dimethyl sulfoxide-d_6_ (DMSO-d_6_, 100%) were received from Sigma-Aldrich. Hydrochloric acid (AR, 35%), KCl (LR, 99.5%), sulfuric acid (LR, 97%), *N*,*N*-dimethylmethanamide (DMF) (HPLC 99.8%), methanol (HPLC, 99.9%), chloroform (AR, 98.8%), methanol (AR, 99.8%) and propan-2-one (AR, 99.8%) were purchased from Rankem, India. Ethanedioic acid (AR, 99%), ceric ammonium nitrate (LR, 98%) and tetrahydrofuran (THF) (AR, 99.5%) were purchased from SD Fine Chemicals, India. Ammonia solution (AR, 25%), copper(ii) chloride dihydrate (LR, 98.8%) and dimethyl sulfoxide (DMSO) (99%) were obtained from Finar, India, and used without further purification.

### 2.2 Chemical synthesis of TAni

Tetraaniline was synthesized *via* a chemical oxidative coupling reaction using different oxidizing agents, namely, ceric ammonium nitrate [(NH_4_)_2_Ce(NO_3_)_6_] and copper(ii) chloride dihydrate [CuCl_2_·2H_2_O] and the emulsion polymerization of dianiline using ammonium peroxodisulfate [(NH_4_)_2_S_2_O_8_], iron(iii) chloride hexahydrate [FeCl_3_·6H_2_O], and benzoyl peroxide.

### 2.3 Ceric ammonium nitrate synthesis of TAni (CAN-TAni)

Ceric ammonium nitrate 1.644 g (3 mmol) was dissolved in 10 mL of 1 M HCl at room temperature. The dimer *N*-phenyl-1,4-phenylenediamine (0.553 g, 3 mmol) was suspended in 20 mL 1 M HCl in a 100 mL beaker with strong magnetic stirring for 0.5 hours at room temperature. The ceric ammonium nitrate solution was added to the dimer suspension very quickly under vigorous stirring. The suspension was then stirred overnight. The resulting material was filtered by vacuum filtration. The collected precipitate was transferred into 30 mL of 1 M HCl. The resulting suspension was stirred for 1 hour and filtered through the same Büchner funnel. This washing process was repeated 5 times. Alternatively, the precipitate could be separated and washed by centrifugation. The precipitate was dedoped with 1 M ammonium hydroxide for 3 hours at room temperature, collected by filtration, washed several times with distilled water, and dried under dynamic vacuum for 24 hours at room temperature. Yield. 0.4 g.

^1^H NMR (500 MHz, DMSO-d_6_, *δ*, ppm): 8.32 (s, 1H), 7.29–7.23 (t, *J* = 7.5 Hz, 2H), 7.16–7.08 (m, 4H), 7.06–6.78 (m, 9H), 6.68–6.58 (m, 2H), 5.52 (s, 2H).

IR (KBr): *ν*_C=C_ (N=Q=N): 1594 cm^−1^, *ν*_C=C_ (N–B–N): 1503 cm^−1^ and ESI MS: [M + H]^+^ 365.

### 2.4 Copper(ii) chloride synthesis of TAni (CC-TAni)

Tetraaniline (TAni) was synthesized *via* oxidative coupling using copper(ii) chloride dihydrate as the oxidizing agent. Copper(ii) chloride dihydrate (0.523 g, 3 mmol) was first dissolved in 10 mL of 1 M HCl at room temperature. *N*-Phenyl-1,4-phenylenediamine (0.553 g, 3 mmol) was suspended in 20 mL of 1 M HCl in a 100 mL beaker with vigorous stirring for 30 min at room temperature to ensure uniform suspension. The copper(ii) chloride dihydrate solution was introduced into the stirred dimer suspension under strong stirring. The reaction mixture was constantly stirred overnight at room temperature. The obtained product was filtered by vacuum filtration and subsequently redispersed in 30 mL of 1 M HCl. The resulting suspension was stirred for an additional 1 h and then filtered through a Büchner funnel. The collected product was washed five times with 1 M HCl to remove residual impurities. Alternatively, the precipitate could be separated and washed by centrifugation. To obtain the dedoped product, the obtained solid product was treated with 1 M ammonium hydroxide for 3 h at room temperature. The blue-colored precipitate was collected by filtration, washed several times with distilled water, and dried under dynamic vacuum for 24 hours at room temperature. Yield. 0.44 g.

^1^H NMR (500 MHz, DMSO-d_6_, *δ*, ppm): 8.38 (s, 1H), 7.26 (t, *J* = 7.5 Hz, 2H), 7.12 (s, 4H), 7.06–6.90 (m, 6H), 6.88–6.79 (m, 3H), 6.64 (d, *J* = 6.8 Hz, 2H), 5.51 (s, 2H); IR (KBr): *ν*_C=C_ (N=Q=N): 1594 cm^−1^, *ν*_C=C_ (N–B–N): 1496 cm^−1^ and ESI MS: [M + H]^+^ 365.

### 2.5 Synthesis of TAni polymerization with APS (APS-TAni)

*N*-Phenyl-1,4-phenylenediamine (1.1054 g, 6 mmol) was dissolved in 75 mL of chloroform in a 250 mL round-bottom flask. Separately, ammonium peroxodisulfate (APS, 1.37 g, 6 mmol) was dissolved in 75 mL of distilled water. The APS solution was added to the dianiline solution under vigorous stirring at room temperature. The resulting two-phase reaction mixture was magnetically stirred for 3 h at room temperature. After stirring, the green precipitate formed in the organic phase. The oxidized tetraaniline was collected by centrifugation, washed several times with double-distilled water, and dried in an oven at 50 °C for 24 h. The resulting tetraaniline was transferred into 1 M ammonium hydroxide solution and stirred for 3 h at room temperature. The resulting blue precipitate (TAni-EB) was collected by vacuum filtration, thoroughly rinsed with distilled water to remove residual ammonia, and dried in an oven at 50 °C for 24 h. Yield: 0.85 g.

^1^H NMR (500 MHz, DMSO-d_6_, *δ*, ppm): 8.38 (s, 1H), 7.38–6.41 (m, 17H), 5.51 (s, 2H);

IR (KBr): *ν*_C=C_ (N=Q=N): 1596 cm^−1^, *ν*_C=C_ (N–B–N): 1505 cm^−1^ and ESI MS: [M + H]^+^ 365.

### 2.6 Synthesis of TAni by polymerization with benzoyl peroxide (BPO-TAni)

*N*-Phenyl-1,4-phenylenediamine (1.84 g, 10 mmol) was dispersed in 65 mL of 1 M H_2_SO_4_ aqueous solution and magnetically stirred for 30 min to obtain a uniform suspension. Benzoyl peroxide (BPO, 2.44 g, 10 mmol) was dissolved in 40 mL of acetone. The BPO solution was added dropwise to the acidic dianiline suspension under vigorous stirring while maintaining an inert (N_2_) atmosphere at room temperature. After the complete addition of BPO, the reaction mixture was continuously stirred for 5 h at room temperature. The obtained green precipitate was collected by vacuum filtration, washed several times with 1 L of double-distilled water, purified by washing with a 2 : 1 acetone : methanol mixture (500 mL : 250 mL), and dried in an oven at 50 °C for 24 h. The obtained oxidized tetraaniline (green precipitate) was treated with 1 M ammonium hydroxide solution for 3 h at room temperature. The resulting blue-colored precipitate (TAni-EB) was collected by vacuum filtration, washed with double-distilled water, and dried in an oven at 60 °C for 24 h. Yield 1.2 g.

^1^H NMR (500 MHz, DMSO-d_6_, *δ*, ppm): 8.16 (s, 1H), 7.81–6.00 (m, 19H); IR (KBr): *ν*_C=C_ (N=Q=N): 1594 cm^−1^, *ν*_C=C_ (N–B–N): 1501 cm^−1^ and ESI MS: [M + H]^+^ 365.

### 2.7 Synthesis of TAni by biphasic polymerization with FeCl_3_·6H_2_O (Fe-TAni)

*N*-Phenyl-1,4-phenylenediamine (DA, 0.553 g, 3 mmol) was dissolved in 50 mL of chloroform in a beaker (250 mL), and 0.81 g of FeCl_3_·6H_2_O (3 mmol) was dissolved in 50 mL of distilled water. The ferric chloride solution was then added slowly to the DA solution along the walls of the beaker. The whole reaction mixture was kept for 24 h at room temperature. After 24 h, a green precipitate formed in the organic layer, which was collected by centrifugation and washed with distilled water. The obtained green precipitate was dedoped with 1 M ammonium hydroxide solution at room temperature for 3 h, filtered, and washed with distilled water. The obtained emeraldine-base TAni was dried in an oven at 50 °C for 24 h. Yield: 0.22 g.

^1^H NMR (500 MHz, DMSO-d_6_, *δ*, ppm): 8.26 (s, 1H), 7.66–6.34 (m, 17H), 5.44 (s, 2H).

IR (KBr): *ν*_C=C_ (N=Q=N): 1593 cm^−1^, *ν*_C=C_ (N–B–N): 1502 cm^−1^ and ESI MS: [M + H]^+^ 365.

The synthesis of the product (tetraaniline) was confirmed using FT-IR spectroscopy and ESI mass spectrometry. The synthetic procedures of tetraaniline are depicted in Scheme 2.

**Scheme 2:**
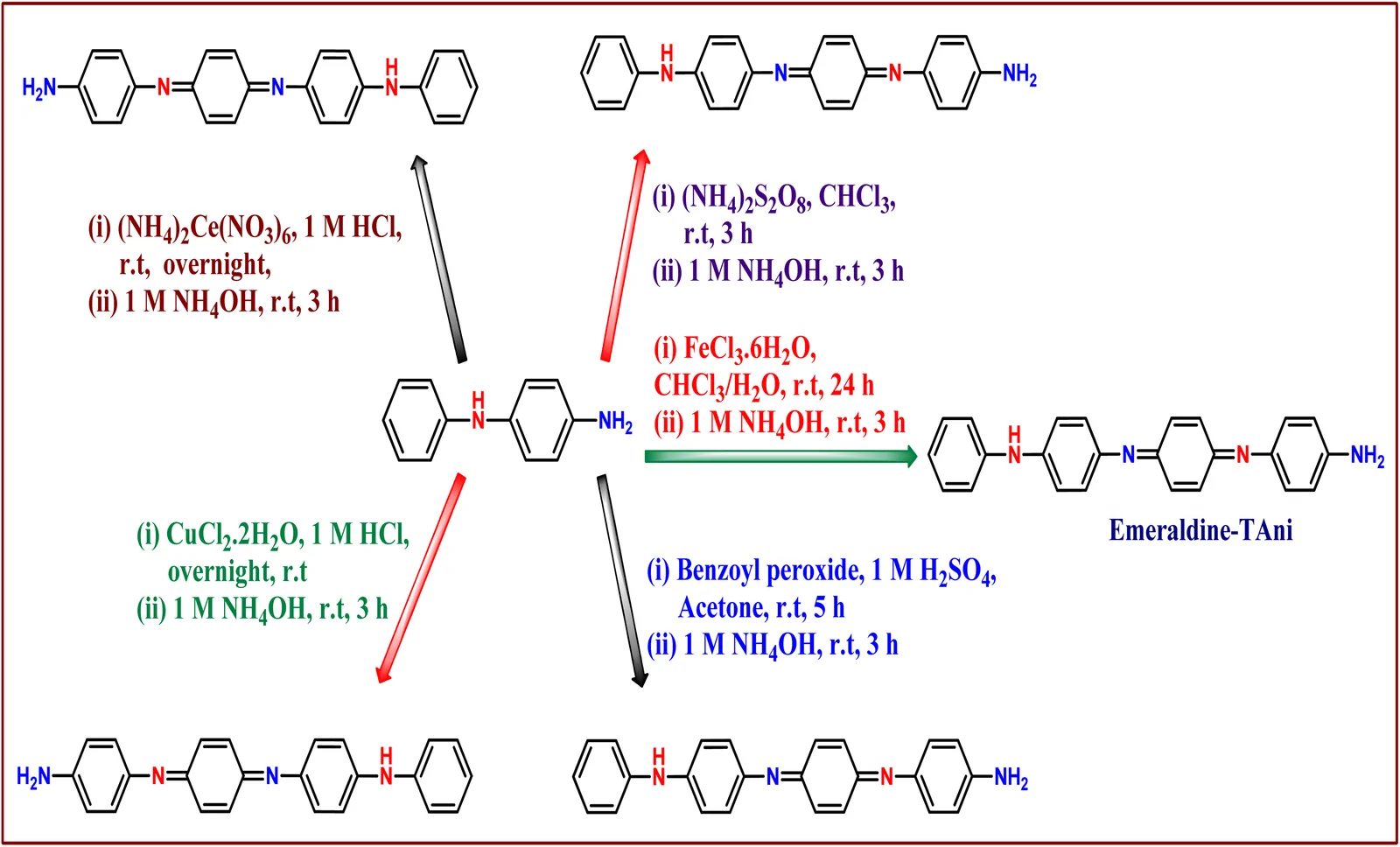
Synthesis of tetraaniline (TAni) using various oxidizing agents.

### 2.8 Pellet making for electronic conductivity

Preliminary protonic doping was carried out on the oxidized tetraaniline samples (CAN-TAni, CC-TAni, APS-TAni, BPO-TAni, and Fe-TAni_bp_). The emeraldine base form of TAni was treated with 1 M hydrochloric acid (HCl) solution at room temperature for 4 h. The resulting green precipitate was collected by filtration and dried in a vacuum oven at 40 °C for 24 h. The dried powder was pressed into pellets with a diameter of 1 cm and a thickness of 1.5 mm using a hydraulic press at an applied pressure of 3 tons.

## 3 Characterization techniques

The molecular structure of the synthesized emeraldine-base tetraaniline samples (CAN-TAni, CC-TAni, APS-TAni, BPO-TAni and Fe-TAni_bp_) was characterized using Fourier-transform infrared (FT-IR) spectroscopy; the spectra were recorded using a PerkinElmer Spectrum (Spectrum 100, PerkinElmer, USA) instrument with the samples prepared as KBr pellets. Each sample was scanned 8 times in the range of 450–4000 cm^−1^. The molecular structures of the synthesized emeraldine-base tetraaniline samples were further characterized using proton nuclear magnetic resonance (^1^H NMR) spectroscopy. The spectra were recorded using a 500 MHz NMR spectrometer (AVANCE-500, Bruker, Switzerland), with the samples dissolved in DMSO-d_6_. ESI mass spectra analysis was conducted using a FINNGAN LCQ Advance Max Instrument; the samples were dissolved in methanol. The UV-vis spectra were recorded using a Shimadzu UV-1800 spectrophotometer (Shimadzu Corporation, Kyoto, Japan) in DMF; the concentration of the samples was on the order of 10^−5^ mol L^−1^. The protonated oligoanilines (TAni-1 M HCl) were pressed into pellets (1 cm diameter, 1.5 mm thickness) under a pressure of 3 tons at room temperature for conductivity measurements. The electrical resistance of all the pellets was measured using the four-probe method employing a Keithley 6220 constant current source coupled with a 2182A (KEITHLEY, Cleveland, Ohio, USA) nanovoltmeter. The thermal stability analysis of the synthesized oligoanilines (CAN-TAni, CC-TAni, APS-TAni, BPO-TAni, and Fe-TAni_bp_) was carried out using thermal gravimetric analysis (TGA) (model. No. Q500, TA Instruments, USA). For this analysis, about 1–5 mg of sample was placed in a crucible and heated at a constant heating rate of 10 °C min^−1^ from room temperature to 700 °C. A constant stream of nitrogen gas (flow rate 30 mL min^−1^) was maintained around the sample chamber to minimize heat dissipation during measurements. Differential scanning calorimetry analyses of the oligomers were performed using a TA Instruments DSC Q100 EXFO Series 2000, USA. For the DSC analysis, samples (wt: 5–10 mg) were hermetically sealed in aluminum pans and heated from −80 °C to 180 °C at a constant heating rate of 10 °C min^−1^ under a liquid nitrogen gas atmosphere (flow rate 30 mL min^−1^). The surface morphology of the oligoaniline powders was examined using field emission scanning electron microscopy (FE-SEM) with a JEOL 7610F, Japan. Oligomer samples were dispersed onto a carbon surface and sputter-coated with a thin gold layer prior to imaging. Electrochemical behavior was investigated *via* cyclic voltammetry (CV) using an AUTOLAB PGSTAT 302N potentiostat/galvanostat in a conventional three-electrode configuration, comprising a saturated calomel electrode (SCE, Hg/Hg_2_Cl_2_) as the reference electrode, a platinum wire as the counter electrode, and a platinum disk as the working electrode. For CV studies, 50 mg of tetraaniline was dissolved in 20 mL of DMF (HPLC grade), and 5 mL of 1 M H_2_SO_4_ aqueous solution was added as a supporting electrolyte. The CV experiment was carried out between −0.2 and 1.0 V at a scan rate of 100 mV s^−1^ at ambient temperature of 25 °C.

## 4. Results and discussion

The chemical synthesis of TAni is only known using the oxidizing agents FeCl_3_(ref. 60) (ref. 60) and H_2_O_2_ (ref. 55) in the literature. No other oxidizing agents have been used. In the present investigation, TAni has been synthesized *via* new chemical routes to study the effect of different routes on the structure and properties of TAni, compared with those of TAni synthesized *via* the standard procedure (Std-TAni) in the presence of FeCl_3_. To achieve enhanced electrical performance, the initially prepared CAN-TAni, CC-TAni, APS-TAni, BPO-TAni, and Fe-TAni_bp_ were subjected to a controlled dedoping–redoping process using aqueous hydrochloric acid. These synthetic strategies, distinct from the established FeCl_3_ and H_2_O_2_ approaches, provide previously unreported access to tetraaniline derivatives and offer new insight into structure–property correlations.

The differences among the TAni samples can be attributed to the distinct redox properties and reaction kinetics of the oxidants. The oxidative coupling of *N*-phenyl-*p*-phenylenediamine likely involves oxidized intermediates followed by coupling and oligomer growth. Variations in the oxidation rate and reactive species generated by CAN, FeCl_3_, APS, CuCl_2_, and BPO can influence chain growth, conjugation, and molecular packing. These effects may subsequently lead to differences in the observed morphology, ranging from compact spherulitic structures to open and porous architectures. The resulting morphology can influence proton-doping accessibility and charge transport, contributing to differences in electrical conductivity. This mechanistic interpretation is based on the observed properties and established redox behavior of the oxidants; detailed kinetic studies would be required for definitive mechanistic confirmation.

### 4.1 Structural characterization of the aniline tetramers

The synthesized aniline tetramer (TAni-EB) structure in its emeraldine base form was characterized using the following techniques: FTIR, proton NMR, ESI-mass, UV-vis absorption spectroscopy and cyclic voltammetry. The corresponding results are presented in Fig. 1–13 and Tables 1–4.

#### 4.1.1 FT-IR spectroscopy

The Fourier-transform infrared (FT-IR) spectroscopy (FT-IR) technique was employed to verify the chemical structure of the synthesized aniline tetramers (TAni). All the samples were scanned in the range of 450 to 4000 cm^−1^. The spectra are shown in Fig. 1 and 2, and the corresponding data are summarized in Table 1.

**Fig. 1:**
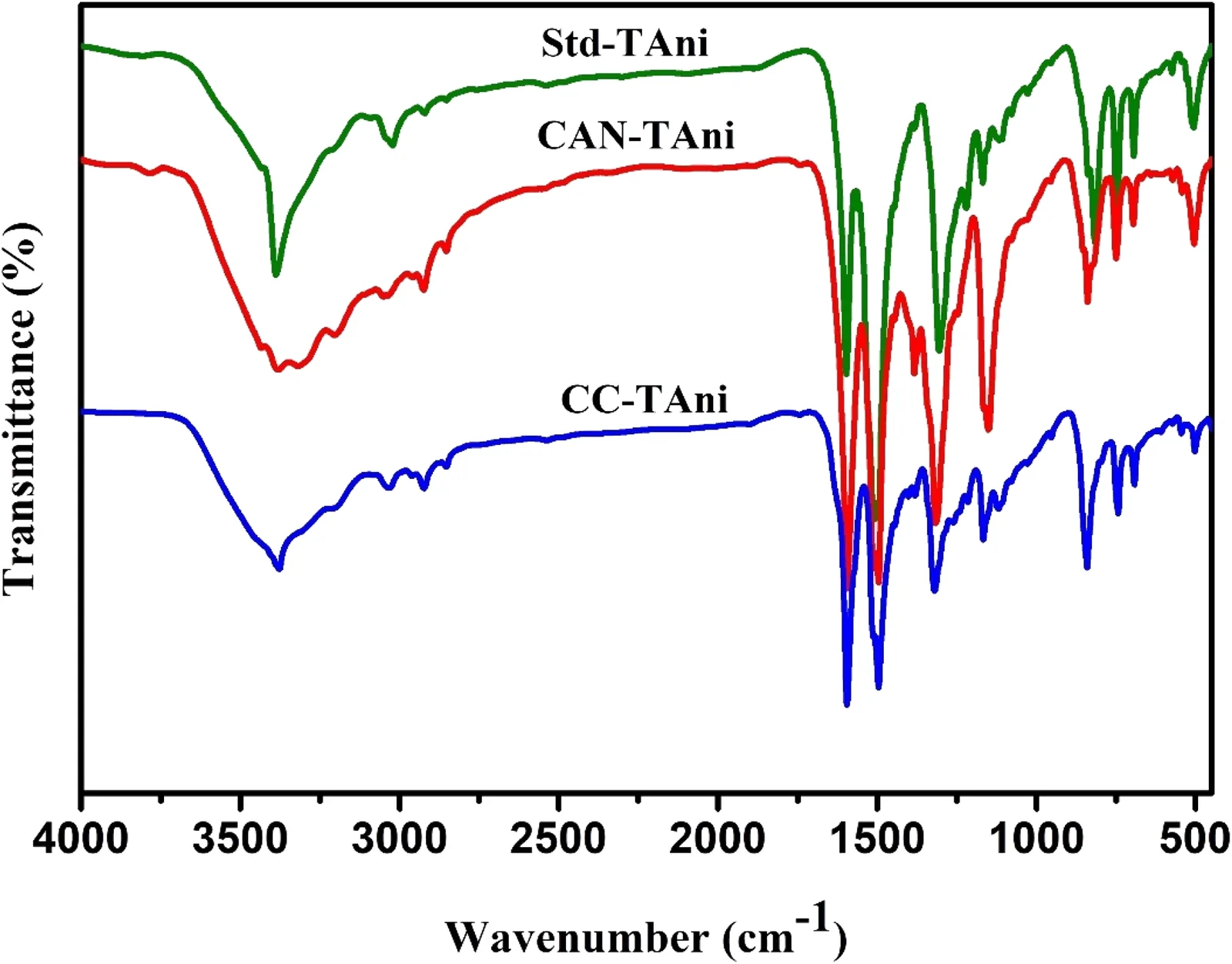
FT-IR spectra of Std-TAni, CAN-TAni and CC-TAni in emeraldine base form.

**Fig. 2:**
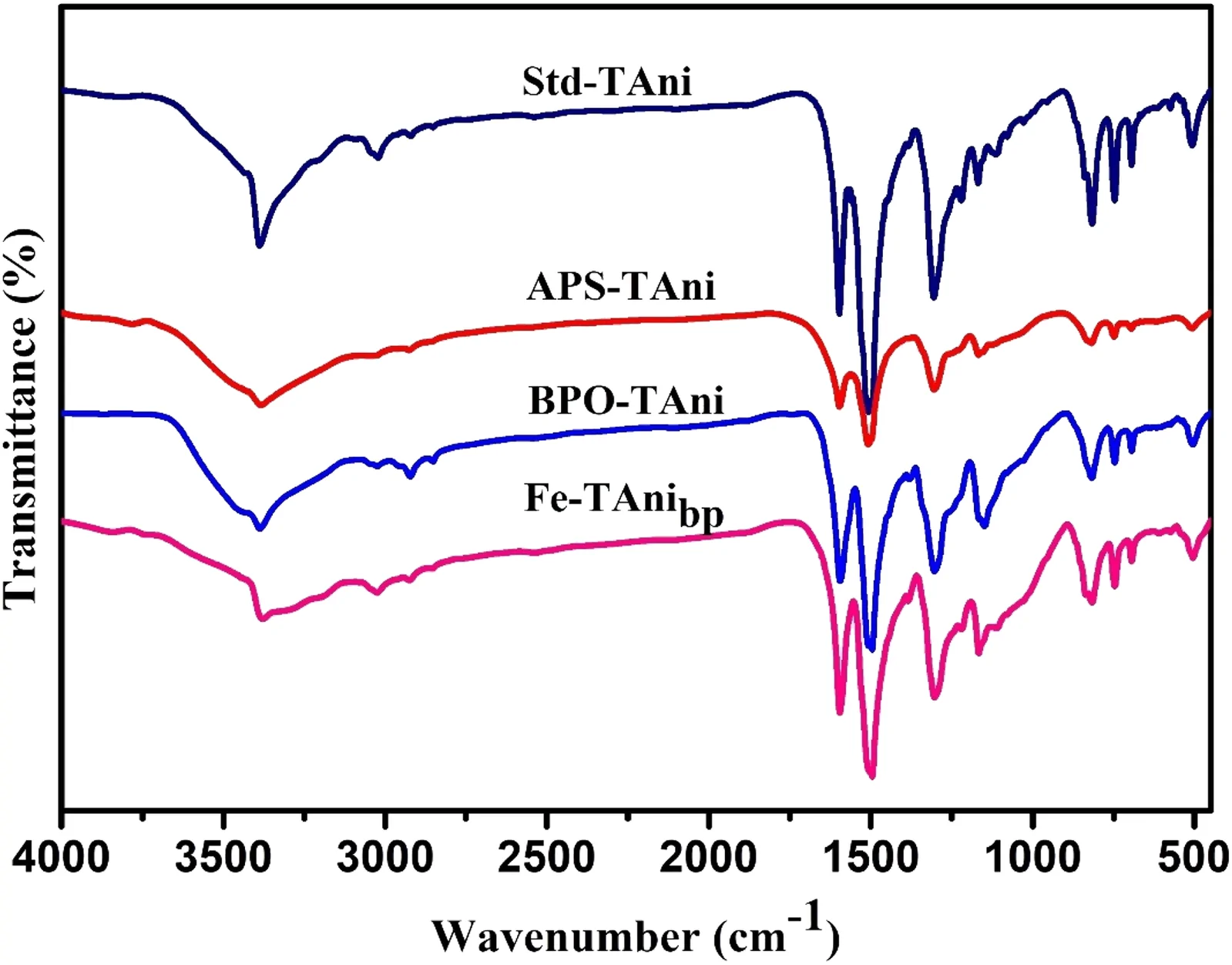
FT-IR spectra of Std-TAni, APS-TAni, BPO-TAni and Fe-TAni in emeraldine base form.

**Table 1:** FT-IR spectral data of CAN-TAni, CC-TAni, APS-TAni, BPO-TAni and Fe-TAni_bp_ in emeraldine base form

	Polymer FT-IR (cm^−1^)						
	C–H_op_	C–N_str_	N–B–N	N=Q=N	C–H_arom_	N–H_str_	–NH_2 str_
CAN-TAni	853	1314	1503	1594	3043	3313	3379
CC-TAni	841	1319	1496	1594	3043	3220	3386
APS-TAni	821	1302	1505	1596	3038	—	3379
BPO-TAni	820	1300	1501	1594	3022	—	3374
Fe-TAni_bp_	818	1300	1502	1593	3022	—	3385

For CAN-TAni and CC-TAni, characteristic absorption bands corresponding to –NH_2_ and –NH– stretching vibrations were observed in the regions 3380–3200 cm^−1^ and 3386–3220 cm^−1^, respectively. In the cases of the other chemical polymerization methods, the obtained tetraaniline showed a single characteristic NH_2_ absorption band at 3379, 3374 and 3385 cm^−1^ for APS-TAni, BPO-TAni, and Fe-TAni_bp_, respectively, while the secondary amine (–NH–) vibration appeared to overlap with the primary amine band, preventing clear resolution. The absorption band in the region 3043–3022 cm^−1^ is attributed to aromatic C–H stretching vibrations of the benzene rings. More importantly, two prominent bands were observed in the aromatic ring region between 1594, 1594, 1596, 1594, 1593 and 1503, 1496, 1505, 1501, 1502 cm^−1^, corresponding to the stretching vibrations of quinoid (C=C and C=N) and benzenoid (C=C) units for CAN-TAni, CC-TAni, APS-TAni, BPO-TAni and Fe-TAni_bp_, respectively.^61–64^ The coexistence of these characteristic vibrations is consistent with the alternating benzenoid–quinoid structure typically observed in aniline oligomers. The presence of the C=N stretching vibration mode indicates the partial oxidation of benzenoid to quinoid structures, indicating successful formation of the emeraldine-type oxidation state of TAni. The absorption band at 1319–1300 cm^−1^ is assigned to C–N stretching vibrations of aromatic amine units.^65,66^ Additionally, the distinct band between 853 and 816 cm^−1^ corresponds to out-of-plane C–H bending of the 1,4-substituted benzene ring, which is a structural signature of head-to-tail coupling in aniline oligomers. The presence of absorption bands in the 750–691 cm^−1^ region (with slight variations depending on the oxidant system) confirms terminal phenyl ring vibrations in the asymmetric tetramer structure. Collectively, these spectral features are consistent with predominantly para-linked aniline units and support the formation of the expected tetraaniline framework.^61^

#### 4.1.2 ^1^H NMR and ESI-mass study

The formation of chemically synthesized emeraldine-base tetraaniline was confirmed *via*^1^H NMR spectroscopy using DMSO-d_6_ as the solvent (Fig. 3 and 4). The observed proton resonances, chemical shifts, and splitting patterns are consistent with the proposed tetrameric structure and confirm the emeraldine-base state. For the samples synthesized using ceric ammonium nitrate (CAN-TAni) and copper chloride (CC-TAni), characteristic signals corresponding to secondary amine (–NH–) and primary amine (–NH_2_) protons were clearly identified. The signals attributed to –NH_*j*_– and –NH_2*i*_ are located at 8.32, 5.52 and 8.38, 5.51 ppm for CAN-TAni and CC-TAni;^67,68^ the presence of these distinct amine signals supports successful oxidative coupling while retaining terminal amine functionalities. The signal of the H_b_ (triplet) protons appears at 7.26 ppm in the spectra of CAN-TAni and CC-Tani, and the H_h_ protons appear at 6.68–6.58 ppm as a multiplet and at 6.64 ppm as a doublet for CAN-TAni and CC-TAni. The peaks at 7.16–7.06 and 7.12 ppm, described as a multiplet and a singlet, are due to the quinoid protons of CAN-TAni and CC-TAni, confirming the coexistence of benzenoid and quinoid units characteristic of emeraldine-base tetraaniline. The characteristic multiplet resonances between 7.06–6.78 and 7.06–6.79 ppm correspond to protons of the substituted benzene rings within the tetramer backbone of CAN-TAni and CC-TAni.^69,70^

**Fig. 3:**
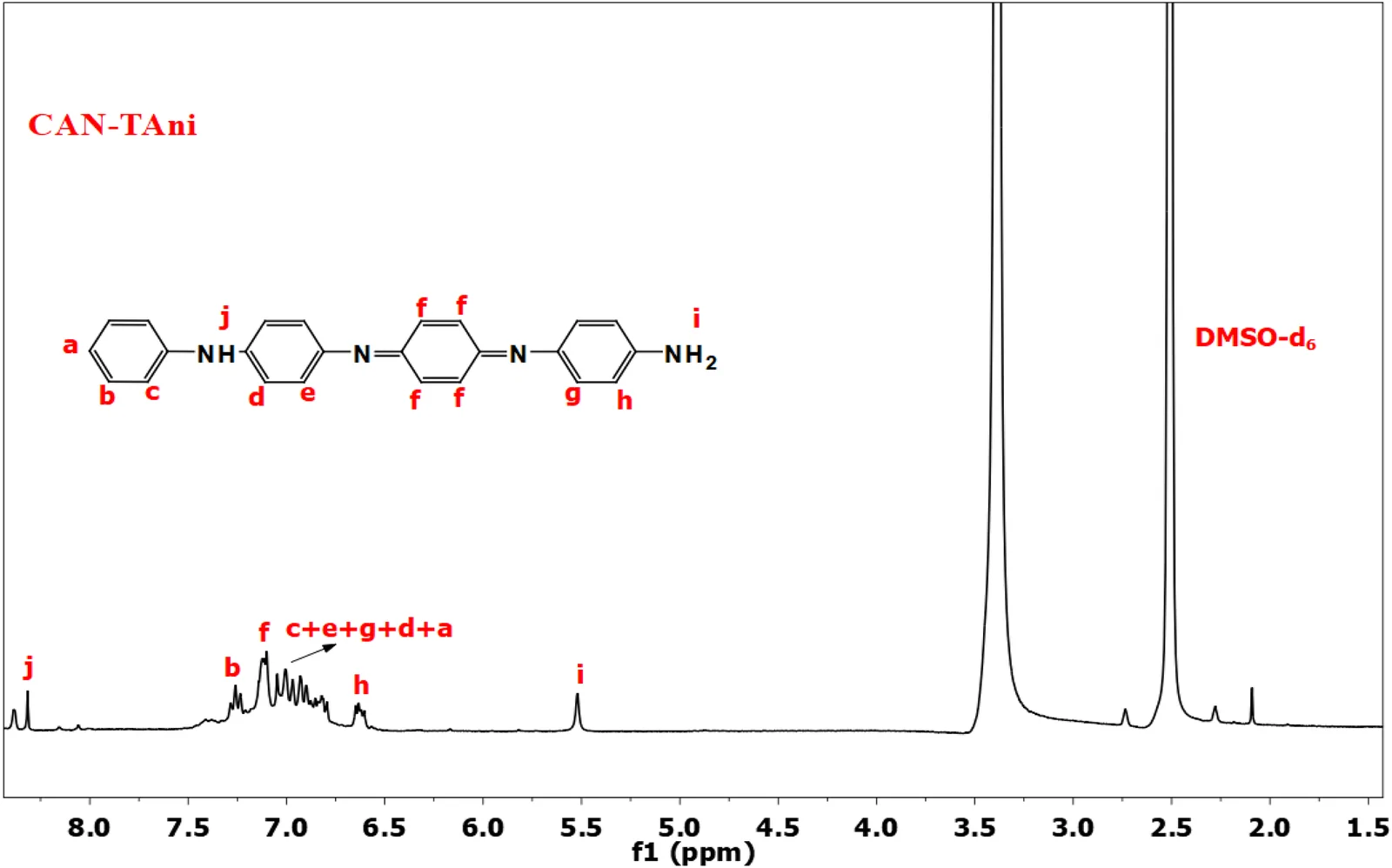
^1^H NMR spectrum of CAN-TAni in DMSO-d_6_.

**Fig. 4:**
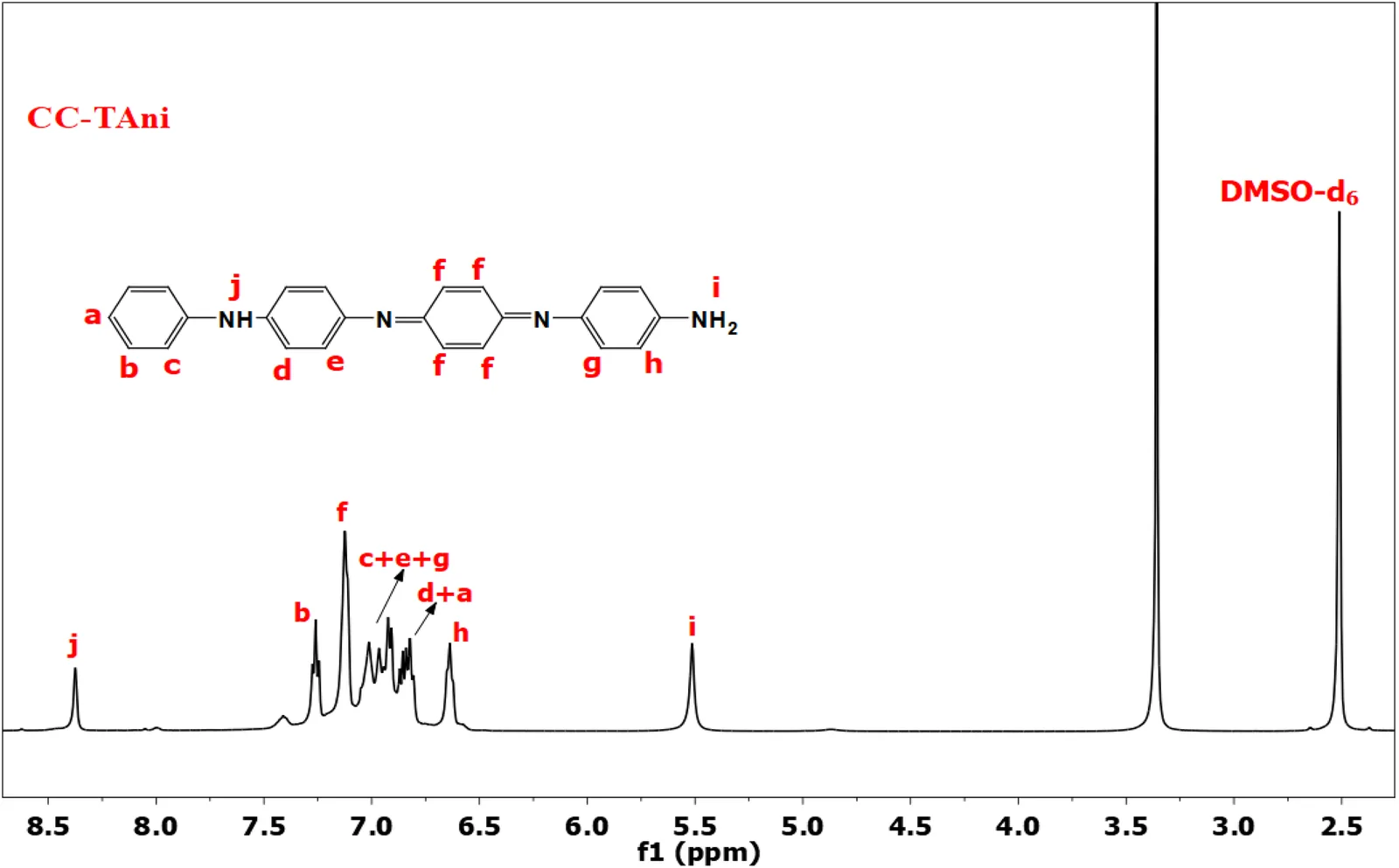
^1^H NMR spectrum of CC-TAni in DMSO-d_6_.

Fig. 5–7 present the ^1^H NMR spectra of the chemically prepared tetramers (PAS-TAni, BPO-TAni and Fe-TAni_bp_); these spectra exhibited comparable structural features. The presence of signals at chemical shift values of 8.38, 5.51 ppm, 8.16 ppm and 8.26, 5.44 ppm attributable to secondary amine (NH_*j*_) and primary amine (NH_2*i*_) was observed for PAS-TAni, BPO-TAni and Fe-TAni_bp_, respectively. Moreover, the characteristic multiplets were observed between 7.38–6.41 ppm, 7.81–6.50 ppm and 7.66–6.34 ppm for PAS-TAni, BPO-TAni and Fe-TAni_bp_, respectively. The overall distribution and integrations of the aromatic signals were consistent with the proposed tetrameric backbone across all preparation methods, indicating preservation of the fundamental conjugated framework. Collectively, the ^1^H NMR data confirm the successful formation of emeraldine-base tetraaniline with the expected benzenoid and quinoid segments. The essentially similar spectra obtained for the different oxidizing systems indicate preservation of the fundamental tetraaniline backbone, while the minor variations in chemical shifts may reflect differences in the local electronic environment and oxidation conditions. Therefore, the ^1^H NMR results do not indicate fundamentally different molecular structures among the samples; rather, the effect of the oxidizing systems on the characteristics of the resulting materials was evaluated based on the combined spectroscopic, morphological, thermal, electrical, and electrochemical analyses.

**Fig. 5:**
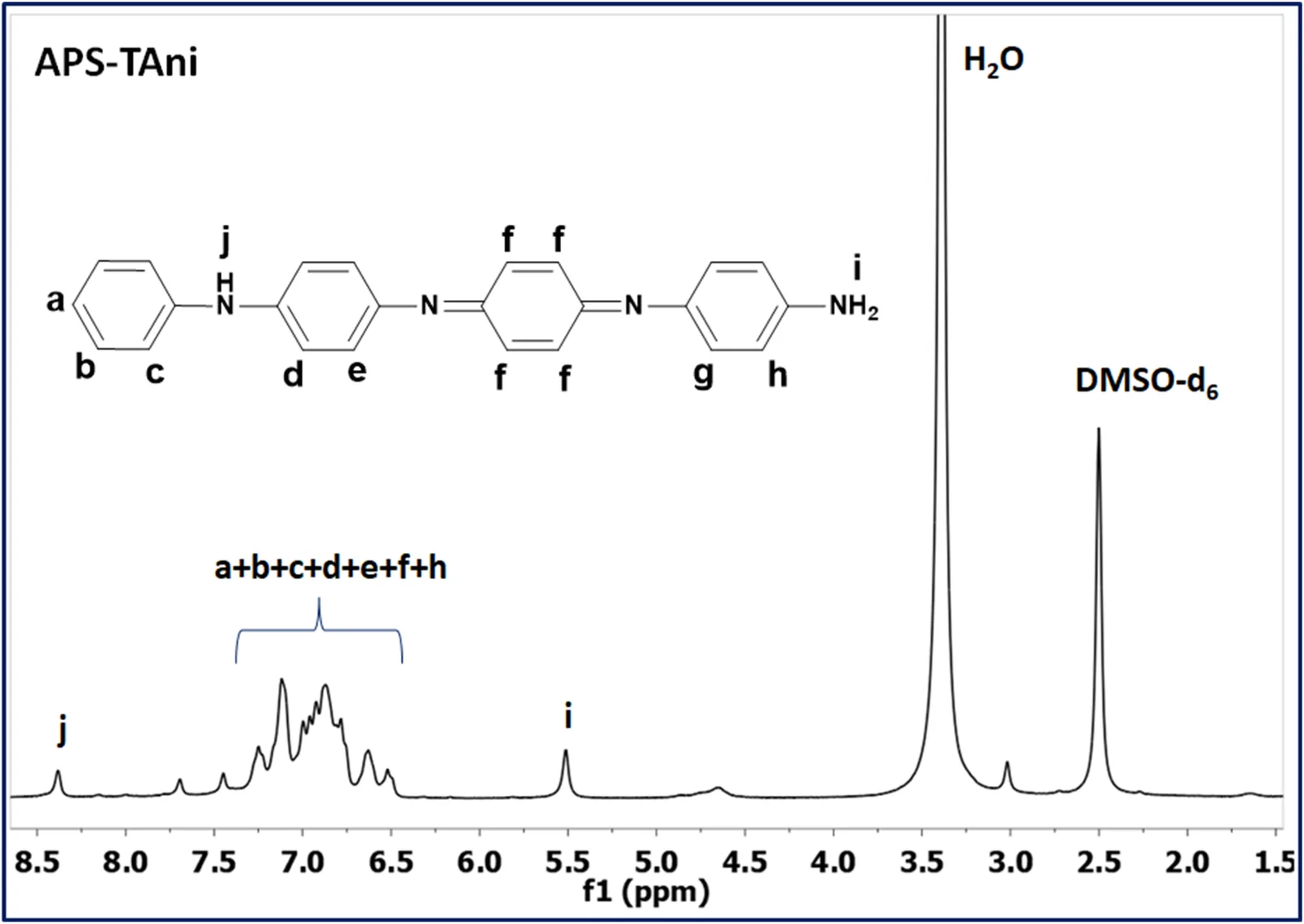
^1^H NMR spectrum of APS-TAni in DMSO-d_6_.

**Fig. 6:**
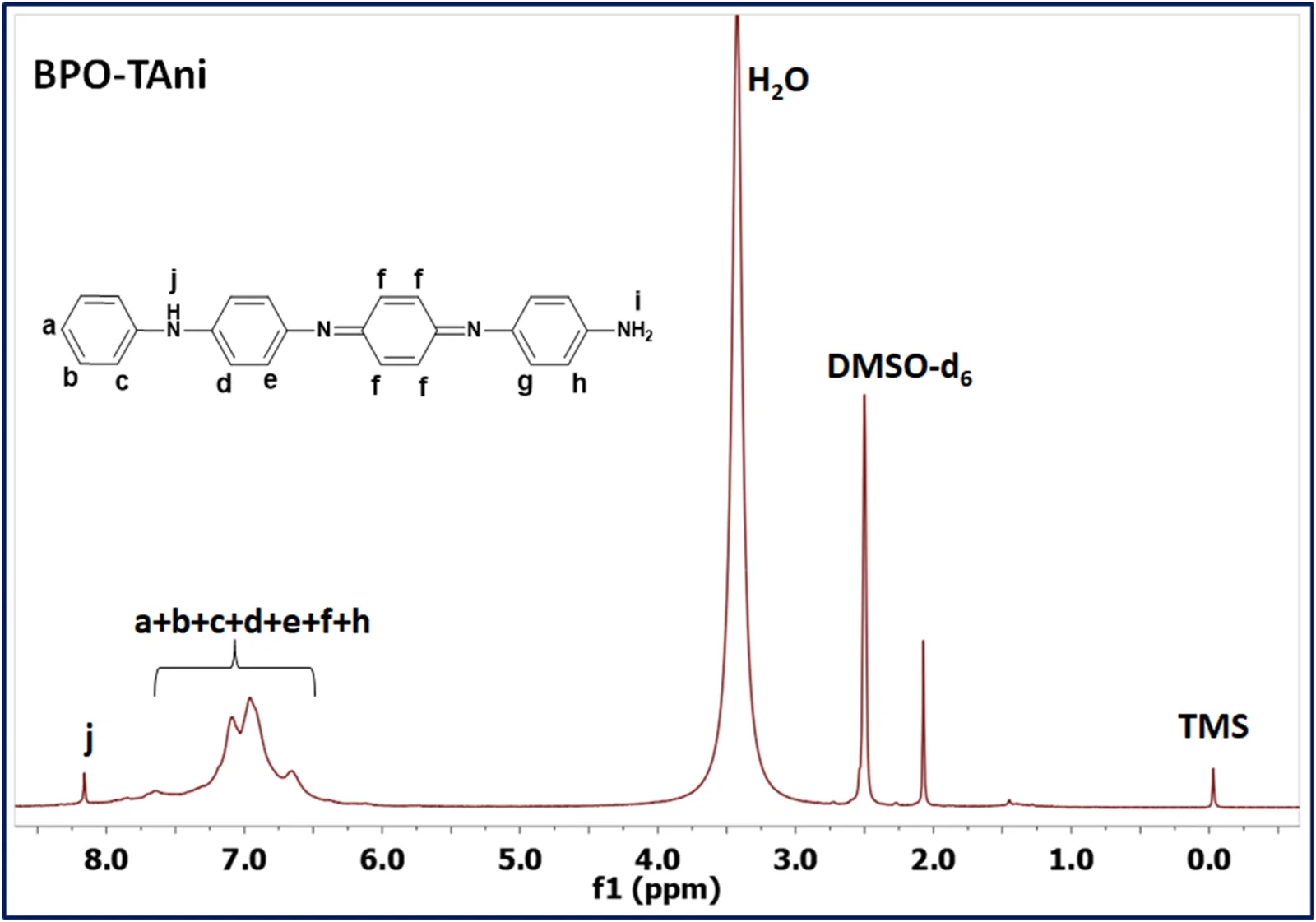
^1^H NMR spectrum of BPO-TAni in DMSO-d_6_.

**Fig. 7:**
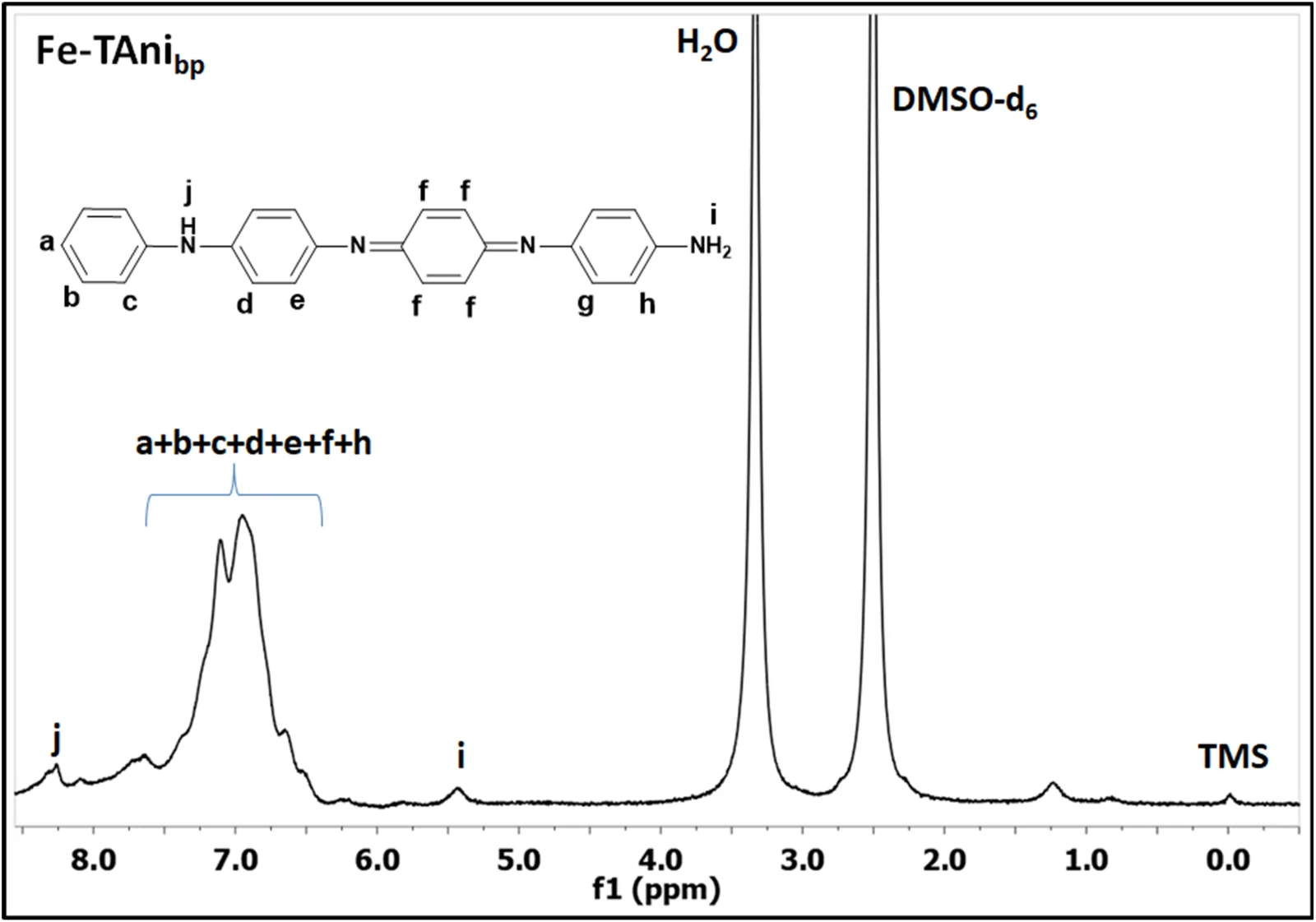
^1^H NMR spectrum of Fe-TAni_bp_ in DMSO-d_6_.

Electrospray ionization mass spectrometry (ESI–MS) was performed to confirm the molecular weight and oligomeric structure of the synthesized tetraaniline (TAni). The spectra of the emeraldine-base (EB) samples (Fig. 8 and 9) exhibited a prominent molecular ion peak at *m*/*z* 365 corresponding to [M + H]^+^, establishing the dimerization of DA to tetraaniline in emeraldine base form. Thus, the ^1^H NMR and mass spectral analyses confirmed the formation of tetraaniline *via* the electrochemical and chemical dimerization methods.

**Fig. 8:**
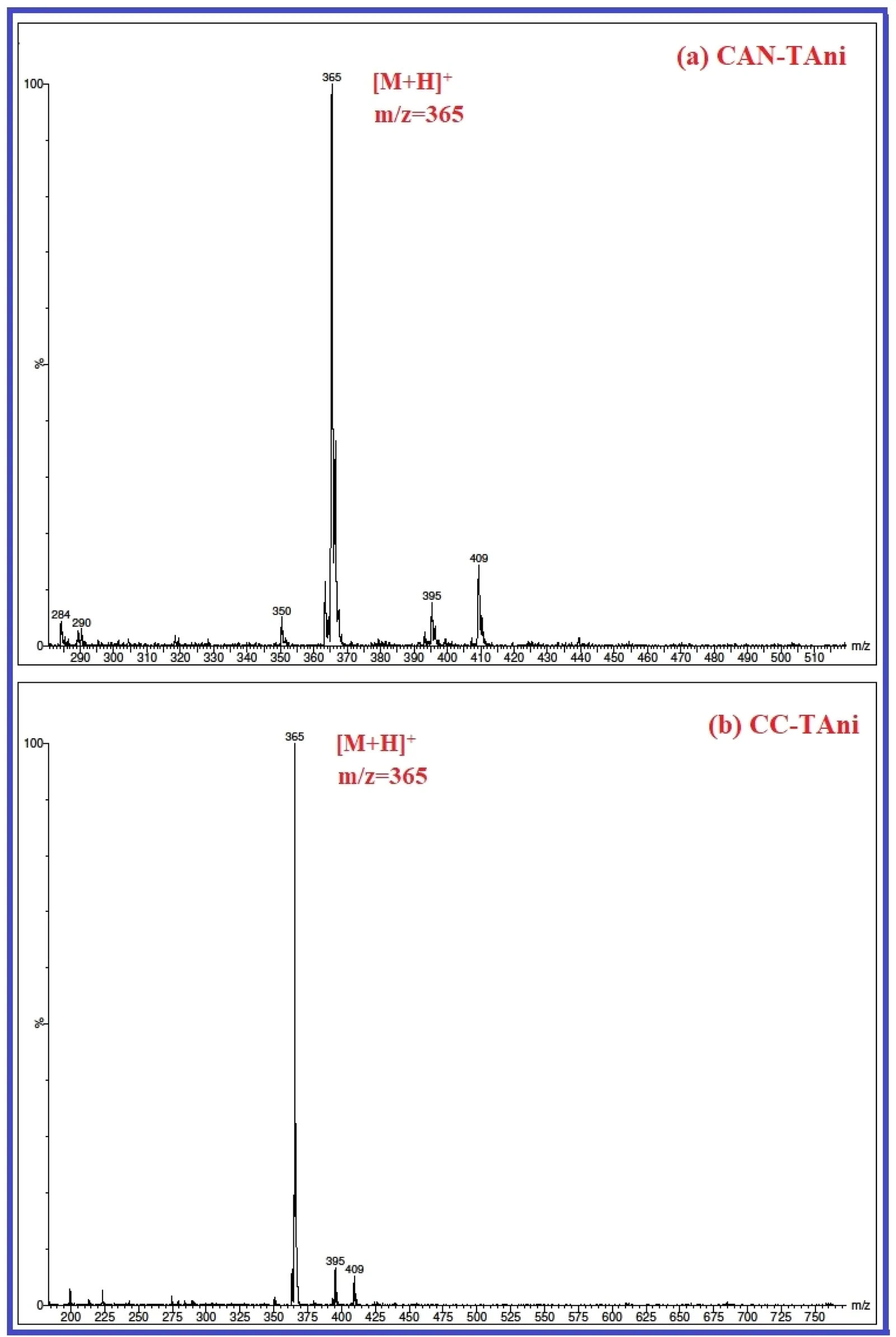
ESI-mass spectra of (a) CAN-TAni and (b) CC-TAni.

**Fig. 9:**
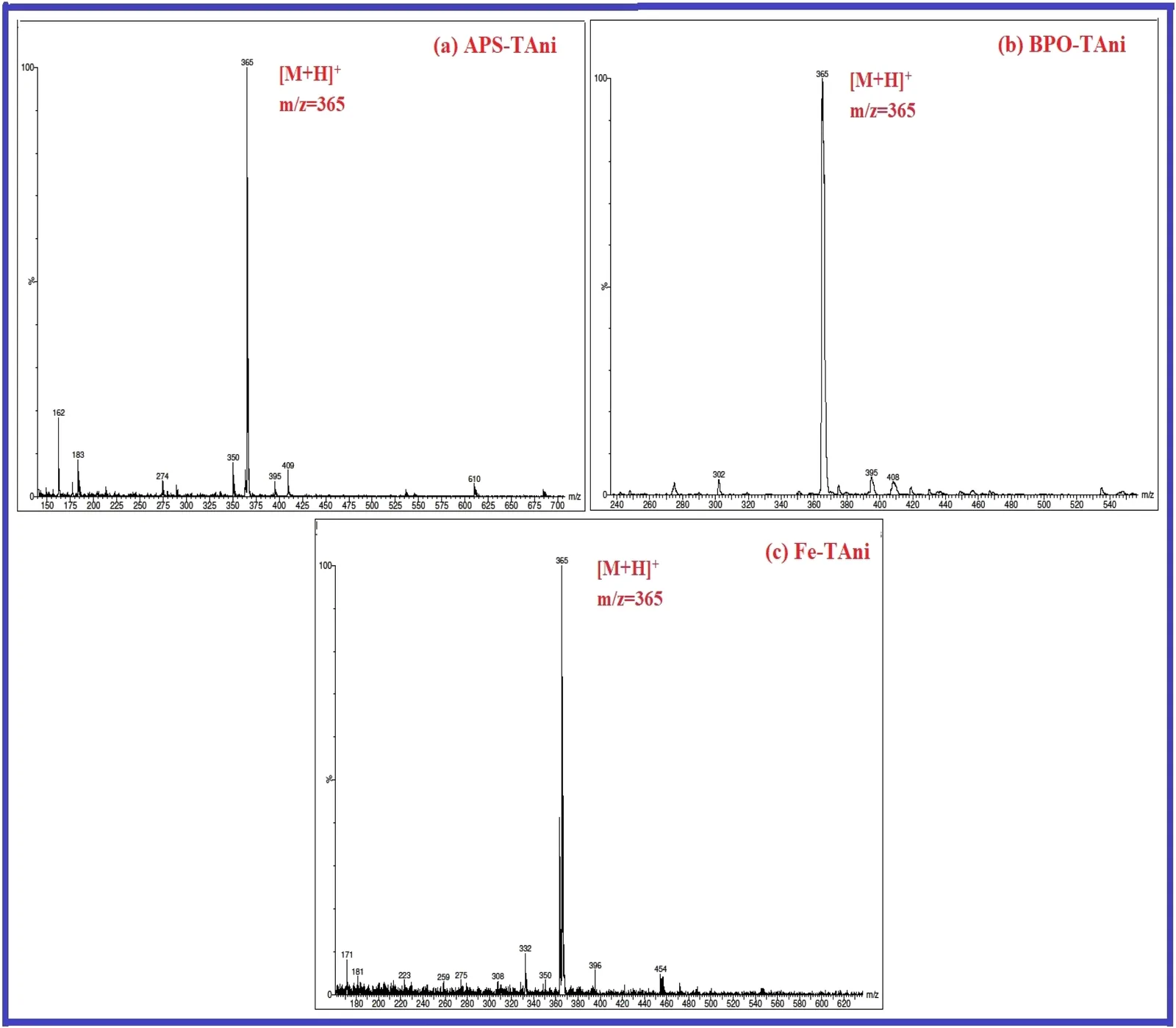
ESI-mass spectra of (a) APS-TAni, (b) BPO-TAni and (c) Fe-TAni_bp_.

Chemically synthesized TAni is typically obtained in its emeraldine base (EB) oxidation state. The conversion of EB to the fully reduced leucoemeraldine base (LEB) form has been reported using reducing agents such as hydrazine or ascorbic acid;^60,71^ however, these approaches generally suffer from limited efficiency and low isolated yields. In addition, side reactions or chain modification may occur when strong reducing agents are employed, potentially affecting structural integrity.^72^

Following reduction, the ESI–MS spectrum of the product (Fig. 10) displayed a molecular ion peak at *m*/*z* 366, consistent with the expected molecular weight of LEB-TAni. The one-unit mass increase relative to EB-TAni reflects the change in oxidation state without alteration of the tetramer backbone, confirming successful reduction rather than chain scission or further oligomerization.

**Fig. 10:**
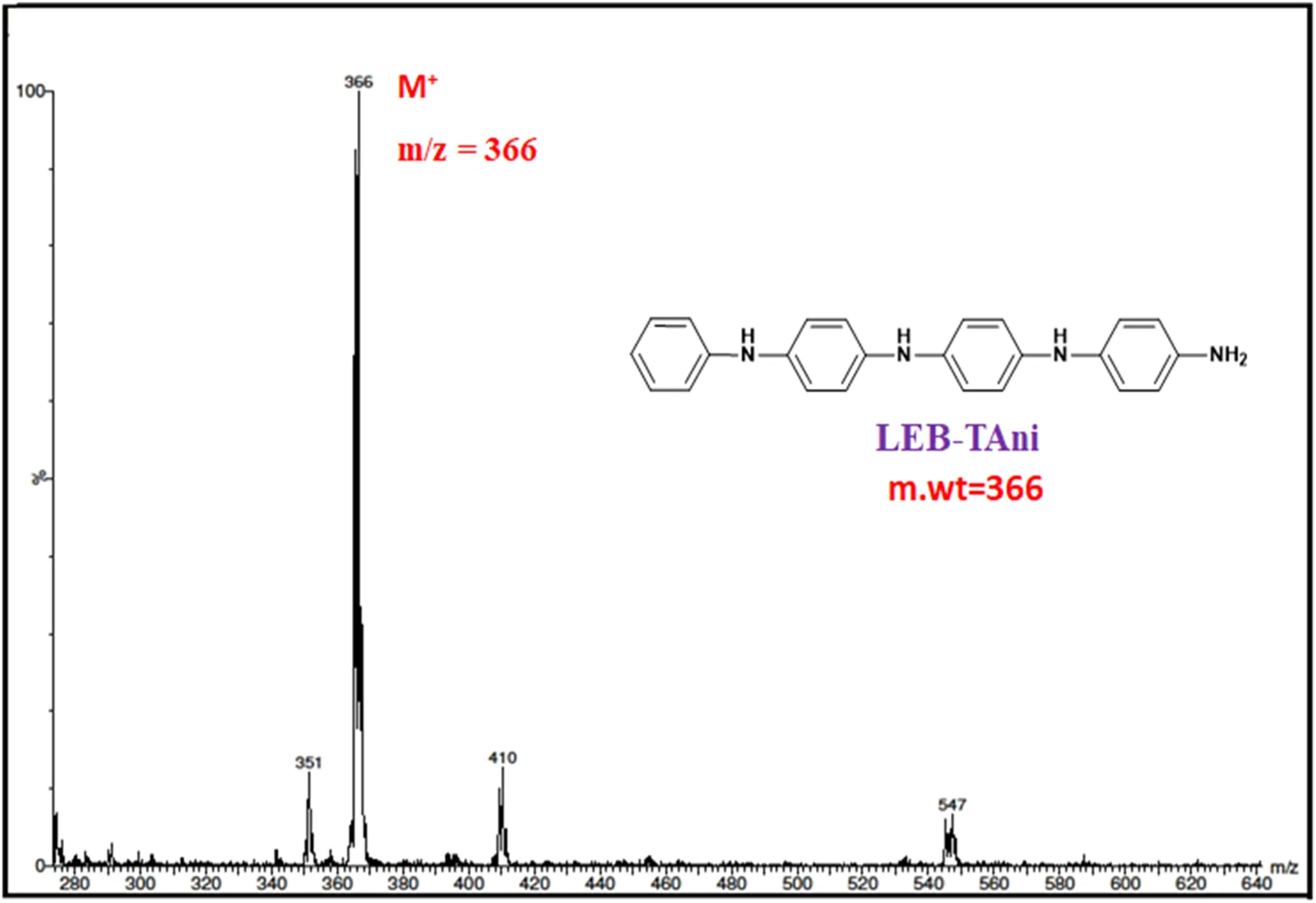
ESI-mass spectrum of LEB-TAni.

The structural features of the reduced tetraaniline were further examined using ^1^H NMR spectroscopy (Fig. 11). The spectrum of LEB-TAni shows well-defined resonances consistent with a fully reduced aromatic framework. The singlet at 4.62 ppm is assigned to terminal primary amine (–NH_2_) protons, confirming preservation of the end groups after reduction. Two additional singlets at 7.44 and 7.68 ppm correspond to secondary amine (–NH_c_–, –NH_d_–) protons within the tetramer chain, while another secondary amine (–NH_b_–) signal overlaps with the aromatic region.^60^ The slight differences in chemical shifts among these –NH– protons can be attributed to variations in their local electronic environments along the conjugated backbone.

**Fig. 11:**
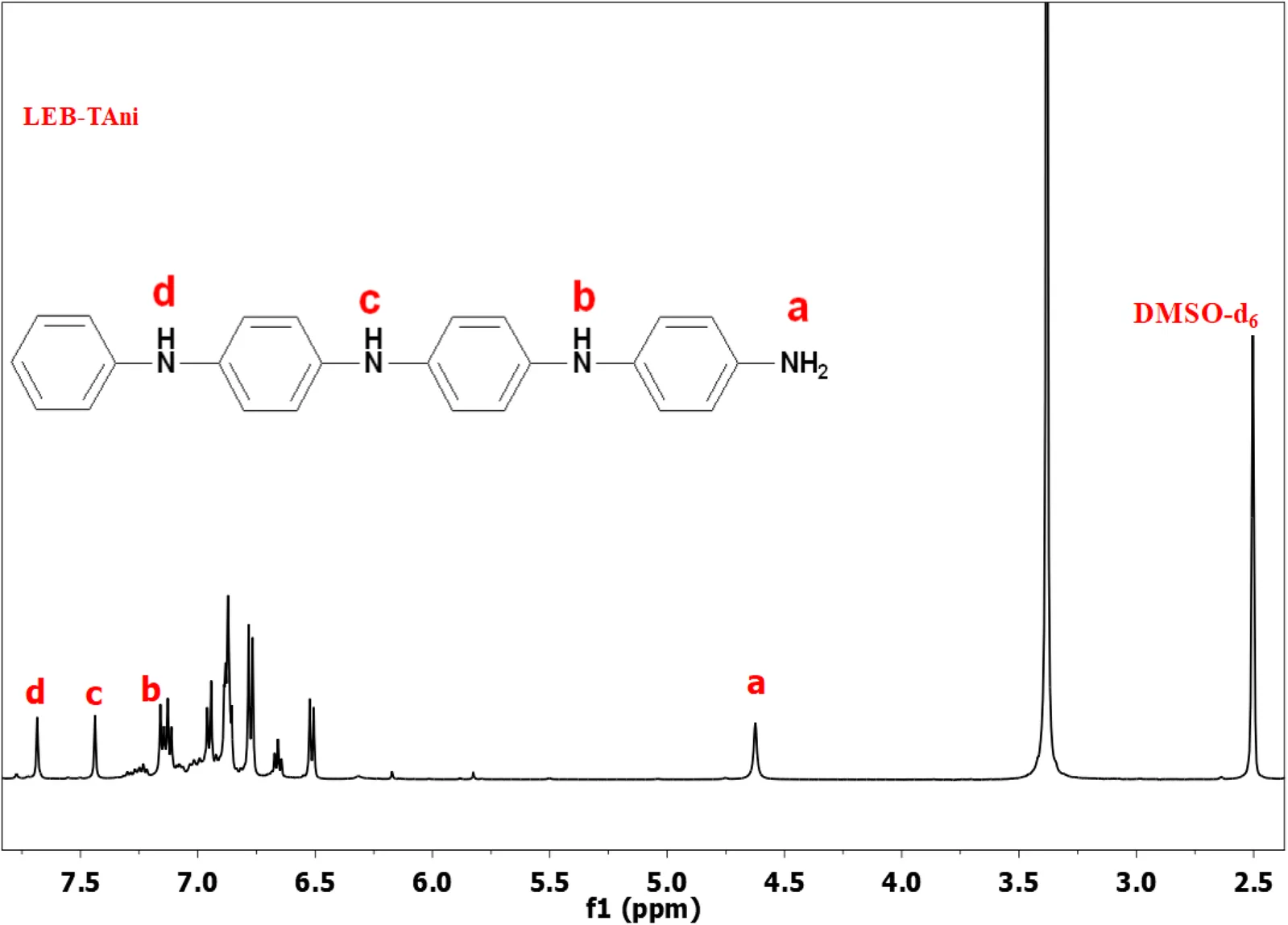
^1^H NMR spectrum of LEB-TAni in DMSO-d_6_.

The doublet at 6.51 ppm and a triplet at 6.66 ppm are assigned to terminal phenyl-ring protons. The signals in the aromatic region between 7.17–6.84 ppm (multiplet) and 6.77 ppm (doublet) correspond to internal phenyl ring protons. Notably, the positions of these aromatic signals remain largely unaffected by chain length, indicating that reduction alters the oxidation state but does not disrupt the fundamental tetrameric structure.^60,73^ Overall, the combined ESI–MS and ^1^H NMR analyses provide clear evidence for the formation of tetraaniline in the emeraldine base state through chemical or electrochemical oxidation, as well as its successful conversion to the leucoemeraldine base form upon electrochemical reduction.

#### 4.1.3 Elemental analysis

The empirical composition of the synthesized aniline tetramer (TAni) samples was determined through CHN elemental analysis using a Vario Micro Cube elemental analyzer. The measured weight percentages of nitrogen, carbon and hydrogen in the samples were compared with those calculated for the theoretically synthesized TAni (Std-TAni).^55^ Elemental analyses were performed for all polymers in their base form, and the experimental data are summarized in Table 2. For all the synthesized samples, the experimentally determined carbon, hydrogen, and nitrogen contents closely matched the theoretical values expected for tetraaniline, confirming the formation of the intended tetrameric structure. Minor deviations between the calculated and experimental values can be attributed to trace solvent retention, adsorbed moisture, or slight variations in oxidation level. Importantly, no significant discrepancies indicative of higher oligomer formation, incomplete coupling, or residual oxidizing agents were observed. Overall, the elemental analysis results corroborate the spectroscopic findings and provide quantitative confirmation of the expected molecular composition. The close agreement between theoretical and experimental values supports the successful synthesis of structurally well-defined tetraaniline in its base form, irrespective of the polymerization method employed.

**Table 2:** Elemental analysis data for Std-TAni, CAN-TAni, CC-TAni, APS-TAni, BPO-TAni and Fe-TAni_bp_ in the emeraldine base state

Compound	Calculated			Found		
	N%	C%	H%	N%	C%	H%
Std- TAni	15.37	79.10	5.53	15.31	78.9	5.61
CAN-TAni	15.37	79.10	5.53	14.93	79.02	5.25
CC-TAni	15.37	79.10	5.53	15.02	78.96	5.52
APS-TAni	15.37	79.10	5.53	15.24	78.99	5.82
BPO-TAni	15.37	79.10	5.53	15.19	78.98	5.47
Fe-TAni_bp_	15.37	79.10	5.53	14.99	78.87	5.35

#### 4.1.4 Optical absorption spectroscopy

UV-Visible electronic absorption spectroscopy is a very important technique in the synthesis of conducting polymers to identify the conducting and insulating states. UV-Vis spectra were recorded in DMF at a concentration of 1 × 10^−5^ mol L^−1^ over the wavelength range 270–900 nm at room temperature (Fig. 12 and 13), and the corresponding absorption maxima are summarized in Table 3. In their undoped form, all TAni samples exhibited two well-defined absorption bands. The lower- and higher-wavelength bands appeared at 309, 563; 304, 579; 302, 578; 314, 576; 317, 582; and 319, 579 nm for Std. TAni, CAN-TAni, CC-TAni, APS-TAni, BPO-TAni and Fe-TAni_bp_, respectively. The higher-energy optical band between 302–321 nm was assigned to π–π* transitions in the conjugated benzene rings of the oligomer backbone.^74,75^ The second optical band recorded between 575–582 nm is attributed to excitonic transitions between benzenoid and quinoid segments (π_B_–π_Q_).^76^ The presence of these two characteristic bands confirms that all samples predominantly exist in the emeraldine base (EB) oxidation state, consistent with the electronic structure reported for chemically synthesized tetraaniline in the literature.^69,77^

**Fig. 12:**
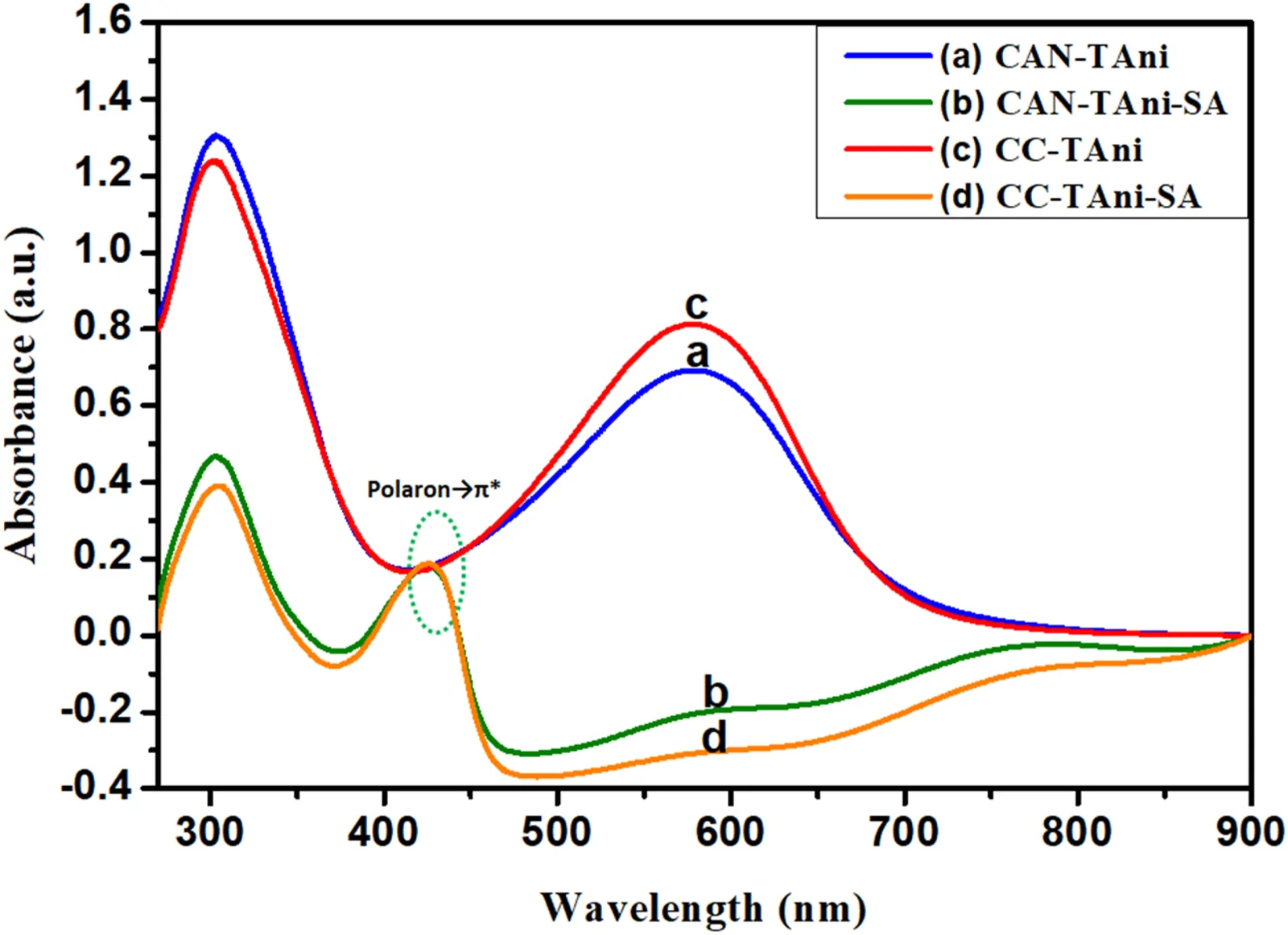
UV-vis spectra of (a) CAN-TAni and (c) CC-TAni in the emeraldine oxidation state in DMF solution, and (b) CAN-TAni-SA and (d) CC-TAni-SA doped *in situ* with 1 M H_2_SO_4_ solution.

**Fig. 13:**
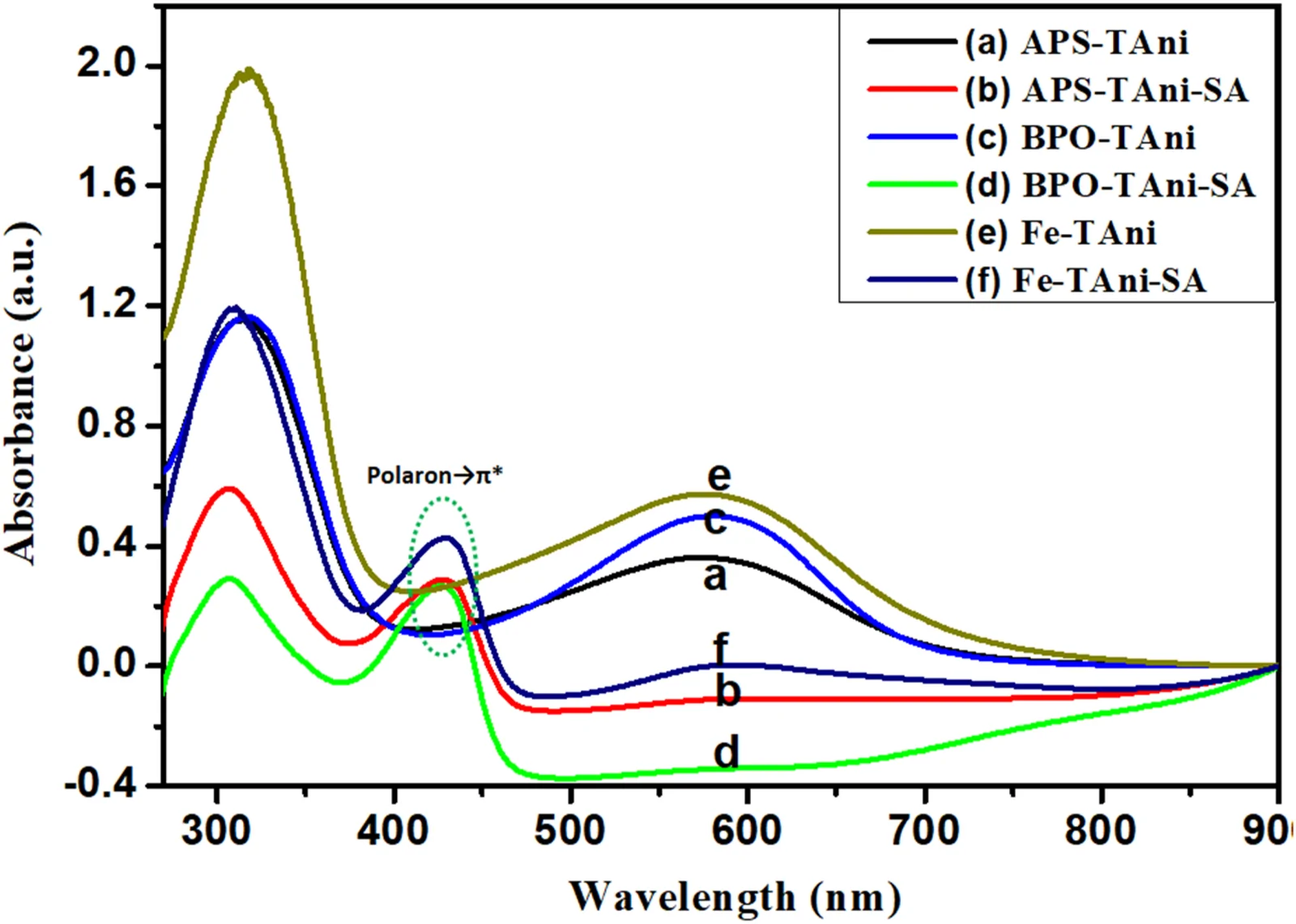
UV-vis spectra of (a) APS-TAni, (c) BPO-TAni and (e) Fe-TAni_bp_ in the emeraldine oxidation state in DMF solution and (b) APS-TAni-SA, (d) BPO-TAni-SA and (f) Fe-TAni_bp_-SA doped *in situ* with 1 M H_2_SO_4_ (SA).

**Table 3:** UV-Vis data of Std-TAni, CAN-TAni, CC-TAni, APS-TAni, BPO-TAni and Fe-TAni_bp_ in the emeraldine base state and doped with 1 M H_2_SO_4_ (SA) solution state

Polymer	UV-vis (DMF) (*λ*_max_) nm
Std-TAni	309, 563
Std-TAni-H_2_SO_4_	308, 423, 645
CAN-TAni	304, 579
CAN-TAni-H_2_SO_4_	303, 425, 589, 778
CC-TAni	302, 578
CC-TAni-H_2_SO_4_	304, 426, 765
APS-TAni	314, 576
APS-TAni-H_2_SO_4_	307, 429, 635
BPO-TAni	317, 582
BPO-TAni-H_2_SO_4_	307, 428, 599
Fe-TAni_bp_	319, 579
Fe-TAni_bp_-H_2_SO_4_	309, 429, 591

Pronounced changes in the UV-Vis absorption spectra were observed for the aniline tetramers upon protonic doping with 1 M sulfuric acid. The solution color shifted from blue to green, indicative of a transformation from the insulating emeraldine base to the conductive emeraldine salt (ES).^78,79^ While the π–π* transition in the 302–321 nm region remained essentially unchanged, reflecting the preservation of the benzenoid framework, two new spectral features emerged. The new band at around 430 nm and the very broad peak at around 800 nm (Fig. 12) were attributed to the delocalized polaron (polaron → π* and π → polaron transition) bands.^69,80^ In the doped state, the most prominent band emerged at 423, 425, 426, 429, 428, and 429 nm for Std.-TAni, CAN-TAni, CC-TAni, APS-TAni, BPO-TAni and Fe-TAni_bp_, respectively (Fig. 12 and 13). This band is associated with polaron–π* transitions arising from the formation of radical cations along the conjugated backbone (quinoid ring).^24,69^ A slight negative absorbance region was observed in some of the H_2_SO_4_-doped TAni spectra, which was attributed to background/baseline effects associated with the strongly altered absorption profile of the protonated samples. This region was not considered to be a genuine spectral feature. Further baseline adjustment to force the absorbance to zero resulted in distortion or loss of the characteristic doped-state absorption bands; therefore, the original spectral treatment was retained to preserve the relevant electronic transitions of the doped TAni samples. The band at around 800 nm was clearly observed for CAN-TAni and CC-TAni, but not for the remaining samples, indicating that the tetramers are in their conductive emeraldine salt form.^80^ The emergence of these polaronic bands confirms the successful protonation and generation of charge carriers characteristic of the conductive emeraldine salt state. The protonation of the oxidized oligomer caused the low-wavelength absorption band to split, while the high-wavelength absorption red-shifted and extended toward the near-IR. The spectra of all tetramers were like that of chemically synthesized tetraaniline, and the absorption peaks shifted slightly to longer wavelengths (Fig. 12). Thus, the UV-Vis peaks of all oligomers are more red-shifted, and the third peak likely shifts to wavelengths longer than 800 nm. Overall, the optical absorption behavior of all synthesized tetramers closely resembles that of standard chemically prepared tetraaniline, demonstrating that the fundamental electronic structure is retained irrespective of the preparation method.

### 4.2 Electrical conductivity of 1 M hydrochloric-acid-doped tetraanilines

The electrical conductivity of the synthesized tetraaniline (TAni) samples was measured after protonation with 1 M hydrochloric acid. The doped powders were pelletized as described in the experimental section, and their room-temperature conductivity was determined using a standard four-probe configuration method. The conductivity behavior of the doped conjugated tetramers was determined by measuring current and voltage responses (hence, resistance), and the conductivity data are presented in Table 4. The electrical conductivity of these samples was determined using the four-probe method as shown in Eq. (1).
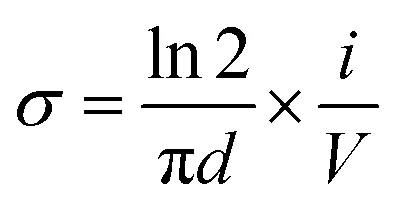
In this technique, a constant current (*I*) is applied through the outer probes, while the voltage drop (*V*) is measured across the inner probes, thereby minimizing contact resistance effects. The surface conductivity (*σ*) was calculated from the measured resistance, considering the pellet thickness (*d*), which was kept smaller than the probe spacing to ensure accurate measurements.

**Table 4:** Conductivity data of protonated chemically synthesized tetraanilines prepared in the presence of 1 M HCl solution at 25 °C

Polymer	Average conductivity (S cm^−1^)
Std-TAni-HCl	3.0 × 10^−3^
CAN-TAni-HCl	1.73 × 10^−1^
CC-TAni-HCl	1.87 × 10^−1^
APS-TAni-HCl	4.7 × 10^−3^
BPO-TAni-HCl	3.32 × 10^−1^
Fe-TAni_bp_-HCl	1.1 × 10^−2^

The doped TAni showed an average conductivity of 1.73 × 10^−1^ and 1.87 × 10^−1^ S cm^−1^ for CAN-TAni and CC-TAni at room temperature. The remaining three synthesized tetraanilines had an average conductivity of 4.7 × 10^−3^, 3.32 × 10^−1^ and 1.1 × 10^−1^ S cm^−1^ for APS-TAni, BPO-TAni, and Fe-TAni_bp_, respectively. As expected, the emeraldine base (EB) form of TAni exhibited insulating behavior (*σ* ≤ 10^−10^ S cm^−1^),^81^ consistent with the absence of mobile charge carriers in the non-protonated state. Among the remaining samples, BPO-TAni-HCl, CC-TAni and CAN-TAni had higher conductivity than other samples because they exhibited a nanostructured morphology with a porous nature. The differences in electrical conductivity among the HCl-doped TAni samples cannot be attributed solely to differences in doping efficiency, as the exact Cl/N ratios were not determined in the present study. The higher conductivity observed for BPO-TAni-HCl may result from a combination of protonation, molecular structure, and morphological characteristics. In particular, the porous nanostructured morphology of BPO-TAni may provide favorable pathways for charge transport and contribute to its higher conductivity compared with APS-TAni-HCl. However, the relative contributions of doping level and morphology cannot be quantitatively separated based on the present data. The conductivity decreases in the order BPO-TAni > CC-TAni > CAN-TAni > Fe-TAni_bp_ > APS-TAni > Std-TAni. These values are better than the 3.0 × 10^−3^ S cm^−1^ reported for chemically prepared Std. TAni in the literature.^55,82^

### 4.3 Thermal analysis of chemically synthesized (CS) tetramers

#### 4.3.1. Thermal gravimetric analysis (TGA)

Thermogravimetric analysis was carried out to evaluate the thermal stability and degradation behavior of the synthesized emeraldine-base tetraaniline (TAni-EB). Thermogravimetric analysis (TGA) gives information about thermal stability, including the degradation kinetics of materials. Measurements were performed under a nitrogen atmosphere from 25 °C to 700 °C at a heating rate of 10 °C min^−1^. The TGA profiles of the electrochemically and chemically synthesized tetramers (CAN, CC, APS, BPO and Fe-TAni_bp_) are presented in Fig. 14(a)–(e). Additionally, the derivative plots for these tetramers are depicted in Fig. 15(a)–(e). The TGA profiles reveal that the CS (CAN, CC, APS, BPO and Fe) emeraldine-base tetramers are thermally stable up to 247 °C. First, a minor weight loss occurs (1.2%) at lower temperatures (<200 °C), which may be attributed to the loss of adsorbed water molecules as well as to other volatile compounds and traces of monomers. Indeed, the derivative plots suggest that all the tetramers show two-step decomposition (Fig. 15(a)–(e)).

**Fig. 14:**
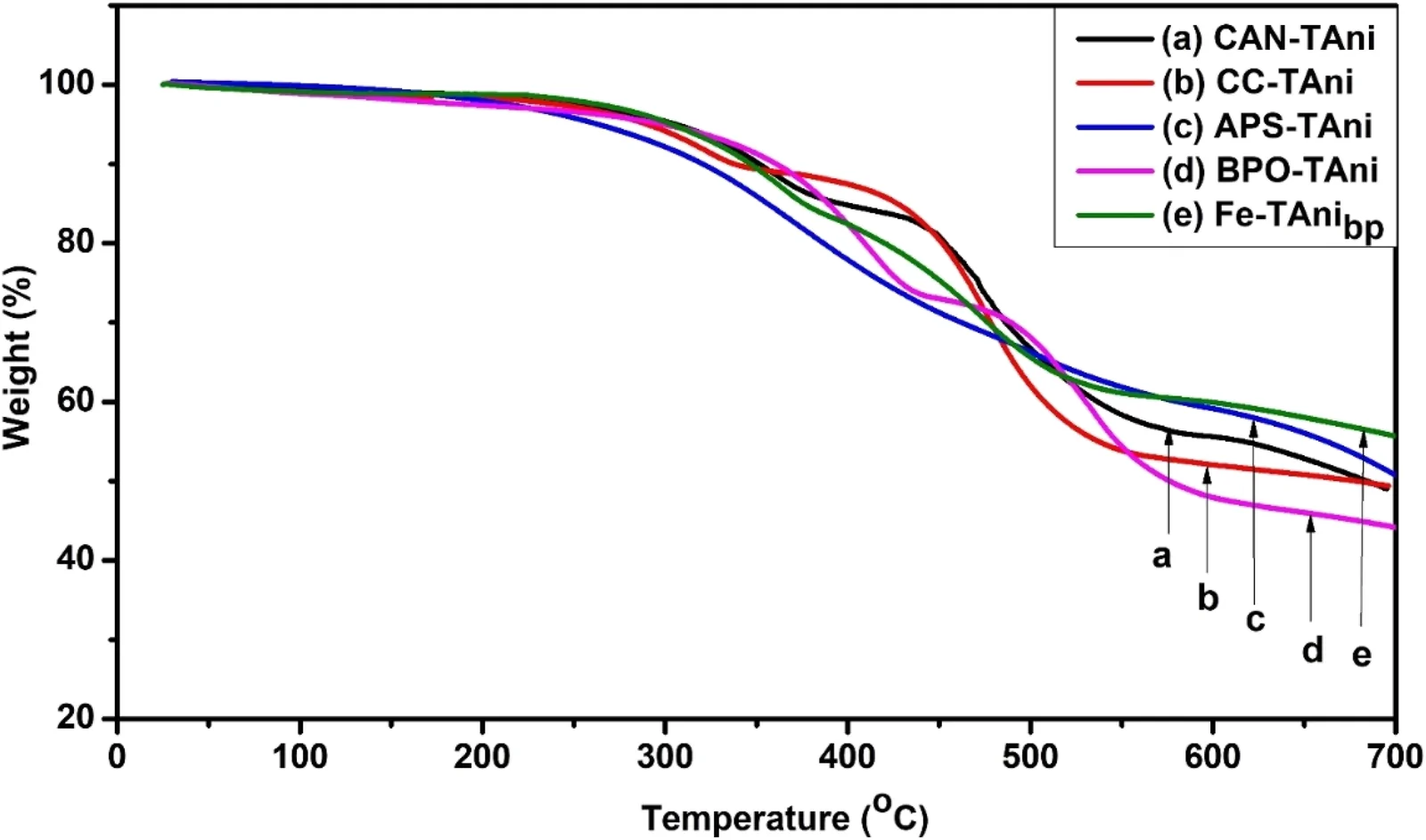
TGA profiles of emeraldine-base tetramers (a) CAN-TAni, (b) CC-TAni, (c) APS-TAni, (d) BPO-TAni and (e) Fe-TAni under a nitrogen atmosphere, at a heating rate of 10 °C min^−1^.

**Fig. 15:**
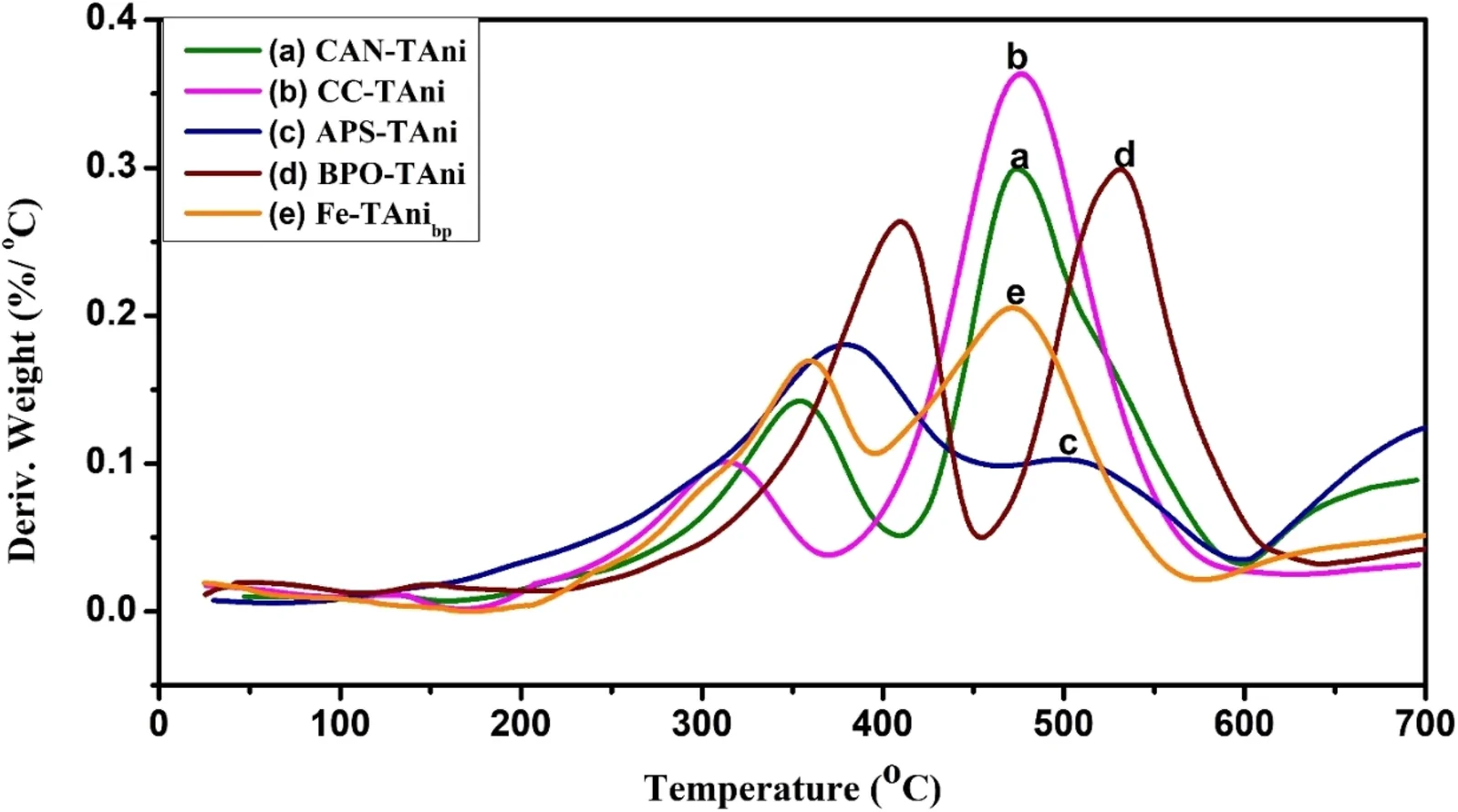
DTG profiles of emeraldine-base tetramers (a) CAN-TAni, (b) CC-TAni, (c) APS-TAni, (d) BPO-TAni and (e) Fe-TAni under a nitrogen atmosphere, at a heating rate of 10 °C min^−1^.

In the first step, the onset of degradation starts (*T*_1ON_) at a temperature of 245.7, 243.8, 247.6, 243.35, 246.8 °C and ends (*T*_1final_) at a temperature around 409.88, 359.59, 440.46, 453.21 and 394.6 °C for CAN-TAni, CC-TAni, APS-TAni, BPO-TAni, and Fe-TAni, respectively. *T*_1max_ values of 353.27, 322, 378.34, 411.86 and 357.16 °C were obtained, respectively. The second decomposition step occurs from 409.88–595.8 °C, 372–570.68 °C, 472–575.8 °C, 453.21–611 °C and 394.6–572.3 °C for the chemically synthesized tetramers, respectively.^35,36^*T*_2max_ values of 474.9, 475.73, 505.49, 531.44 and 474.73 were obtained for the CS tetramers, respectively. The first degradation step is primarily associated with breakdown of the conjugated tetramer backbone and cleavage of amine linkages.^62,80^ The remaining weight percentage at 600 °C is 55.6%, 52.07%, 59.2%, 48% and 59.9% for CAN-TAni, CC-TAni, APS-TAni, BPO-TAni, and Fe-TAni_bp_, respectively. These results indicated that the emeraldine-base tetraaniline is more thermally stable in a nitrogen atmosphere. The data are shown in Table 5.

**Table 5:** TGA data of electrochemically and chemically synthesized emeraldine-base tetramer powder

Polymer	*T*_1ON_ (°C)	*T*_1final_ (°C)	*T*_1max_ (°C)	*T*_2ON_ (°C)	*T*_2final_ (°C)	*T*_2max_ (°C)	Remaining wt% at 600 °C
CAN-TAni	245.7	409.88	353.27	409.88	595.8	474.9	55.6
CC-TAni	243.8	359.59	322	372	570.68	475.73	52.07
APS-TAni	247.6	440.46	378.34	472	575.8	505.49	59.2
BPO-TAni	243.35	453.21	411.86	453.21	611	531.44	48
Fe-TAni_bp_	246.8	394.6	357.16	394.6	572.3	474.73	59.9

#### 4.3.2 Differential scanning calorimetry (DSC)

Differential scanning calorimetry was employed to investigate the thermal transition behavior of the emeraldine-base tetraaniline (TAni-EB) samples. Measurements were carried out under a nitrogen atmosphere over the temperature range of −80 °C to 180 °C. The DSC thermograms are presented in Fig. 16(i) and (ii), and the corresponding transition temperatures are summarized in Table 6. All tetramers exhibited a single, well-defined glass transition temperature (*T*_g_), indicating that each sample behaves as a homogeneous phase without detectable phase separation. The observed *T*_g_ values were 90.68 °C (CAN-TAni), 89.78 °C (CC-TAni), 90.17 °C (APS-TAni), 91.65 °C (BPO-TAni), and 95.78 °C (Fe-TAni).^35^ The narrow range of *T*_g_ values suggests that the chain rigidity and segmental mobility are largely independent of the oxidative route used for synthesis. The slightly higher *T*_g_ observed for Fe-TAni may reflect stronger intermolecular interactions or a marginally more rigid backbone structure. In addition to the glass transition, distinct endothermic thermal events, designated as TM1 and TM2, were observed in the DSC thermograms. As neither TGA-MS nor modulated DSC measurements were performed in the present study, the exact origin of these transitions cannot be conclusively established as melting, desolvation, or other structural relaxation processes. Therefore, these features are described more cautiously as endothermic thermal events rather than being definitively assigned to melting transitions. Among these tetramers, Fe-TAni_bp_ exhibited two melting points at *T*_M1_ = 128.42 °C and *T*_M2_ = 154.53 °C. The remaining tetramers, *viz*. CAN-TAni, CC-TAni, APS-TAni, and BPO-TAni showed one melting point at *T*_M1_ = 148, 142, 153.46, and 139.88 °C, respectively. The presence of sharp melting events indicates a certain degree of structural ordering or crystallinity within the tetramer framework. The appearance of two melting transitions suggests the coexistence of domains with different packing arrangements or crystalline fractions, which may arise from variations in molecular conformation or intermolecular stacking interactions. Overall, the results confirm that emeraldine-base tetraaniline exhibits well-defined thermal transitions characteristic of low-molecular-weight conjugated oligomers and has good thermal stability in a nitrogen atmosphere.

**Fig. 16:**
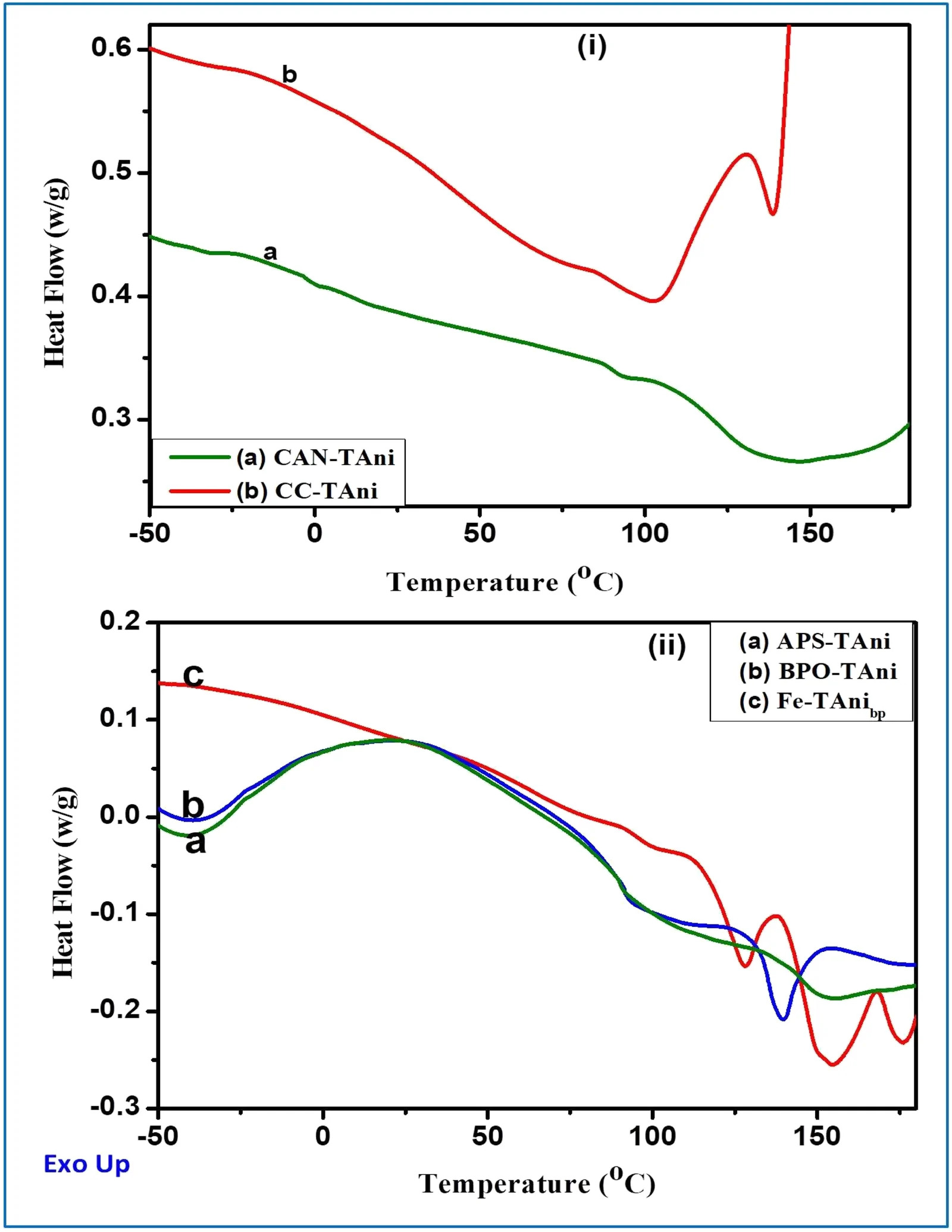
DSC profiles of emeraldine-base tetramers: (i) (a) CAN-TAni and (b) CC-TAni and (ii) (a) APS-TAni, (b) BPO-TAni and (c) Fe-TAni_bp_ under a nitrogen atmosphere, at a heating rate of 10 °C min^−1^.

**Table 6:** DSC data of emeraldine-base tetramers: CAN-TAni, CC-TAni, APS-TAni, BPO-TAni and Fe-TAni_bp_^a^

Polymer	*T*_g_ (°C)	*T*_M1_ (°C)	*T*_M2_ (°C)
CAN-TAni	90.68	148	
CC-TAni	89.78	142	
APS-TAni	90.17	153.46	
BPO-TAni	91.65	139.88	
Fe-TAni_bp_	95.78	128.42	154.53

a *T*_g_ = glass transition temperature, *T*_M1_ = melting temperature 1 and *T*_M2_ = melting temperature 2.

### 4.4 Morphological studies

The surface morphology of the emeraldine-base tetraaniline (TAni-EB) samples was examined *via* field-emission scanning electron microscopy (FE-SEM) using a JEOL 7610F instrument. π-Conjugated oligomers are known to organize into distinct supramolecular architectures through noncovalent interactions such as π–π stacking and hydrogen bonding. Therefore, morphological analysis provides valuable insight into molecular packing and its possible influence on charge transport behavior. The surface morphology of the chemically synthesized tetramers CAN-TAni and CC-TAni, as observed by the FE-SEM, is shown in Fig. 17(i) and (ii), respectively, at various magnifications. Both these tetramers showed a flake-like structure. CAN-TAni displayed a spherulite-like morphology with a characteristic particle size of approximately 1 µm at magnifications of 5k, 10k, and 20k. At higher magnification, corresponding to a 100 nm scale, a nearly disk-like morphology was observed (Fig. 17i(d)), revealing finer structural features. The morphology of CC-TAni at the microscale (10k magnification) is close to spherulites, but when the resolution is increased from the microscale to the nanoscale, it displays a closely stacked granular structure (Fig. 17ii(f–h)).

**Fig. 17:**
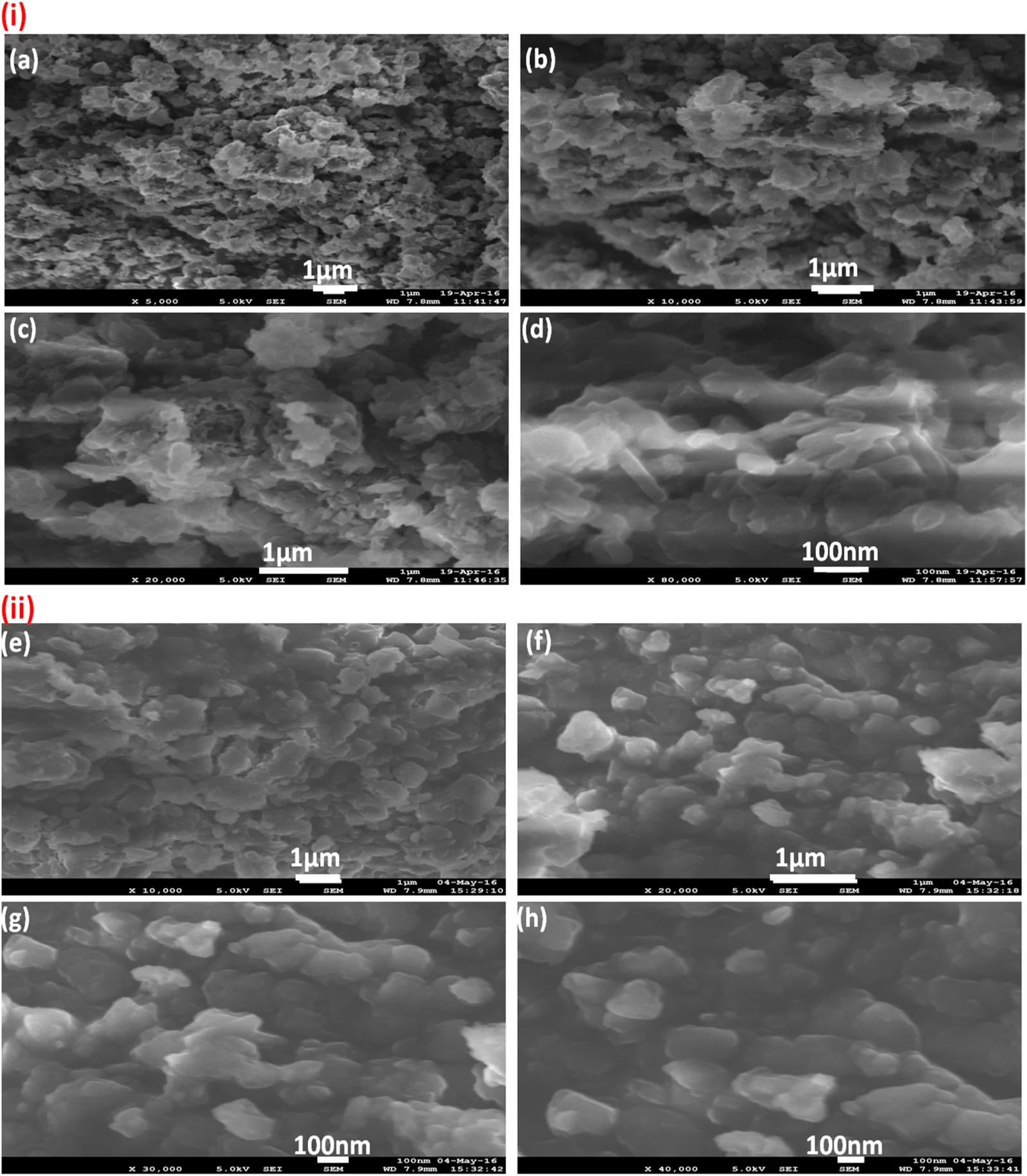
Field emission scanning electron micrograph (FE-SEM) images of emeraldine-base (i) CAN-TAni and (ii) CC-TAni at various magnifications.

Similarly, the morphology of APS-TAni shows a spherulite-like appearance at 3.5k, 5k, and 10k magnifications, and these spherulites show a crystalline nature with irregular shape at the microscale (Fig. 18i(a–c)). BPO-TAni showed a flake-like structure at 10k and 20k magnifications, but at 30k magnification, it appears as an open-sheet-type structure (Fig. 18ii(f)), demonstrating a nanostructured porous material. The morphology of Fe-TAni appeared as flower and flake structures at 10 to 20k magnifications, and was clearly identified at higher (100 nm, 40k) magnification (Fig. 18iii(g–i)). Overall, two general morphological trends can be identified. BPO-TAni and Fe-TAni exhibit more open, porous, and nanostructured morphologies, whereas CAN-TAni, CC-TAni, and APS-TAni display comparatively well-defined, spherulitic features. Overall, the chemically synthesized tetramers exhibited nanostructured morphologies with a more porous/open structure than the electrochemically prepared samples. The nanostructured morphology of the former samples is responsible for their higher conductivity compared to the ECS samples. Due to their nanostructure and porosity, doping is effective, and hence, conductivity increases (Fig. 19).

**Fig. 18:**
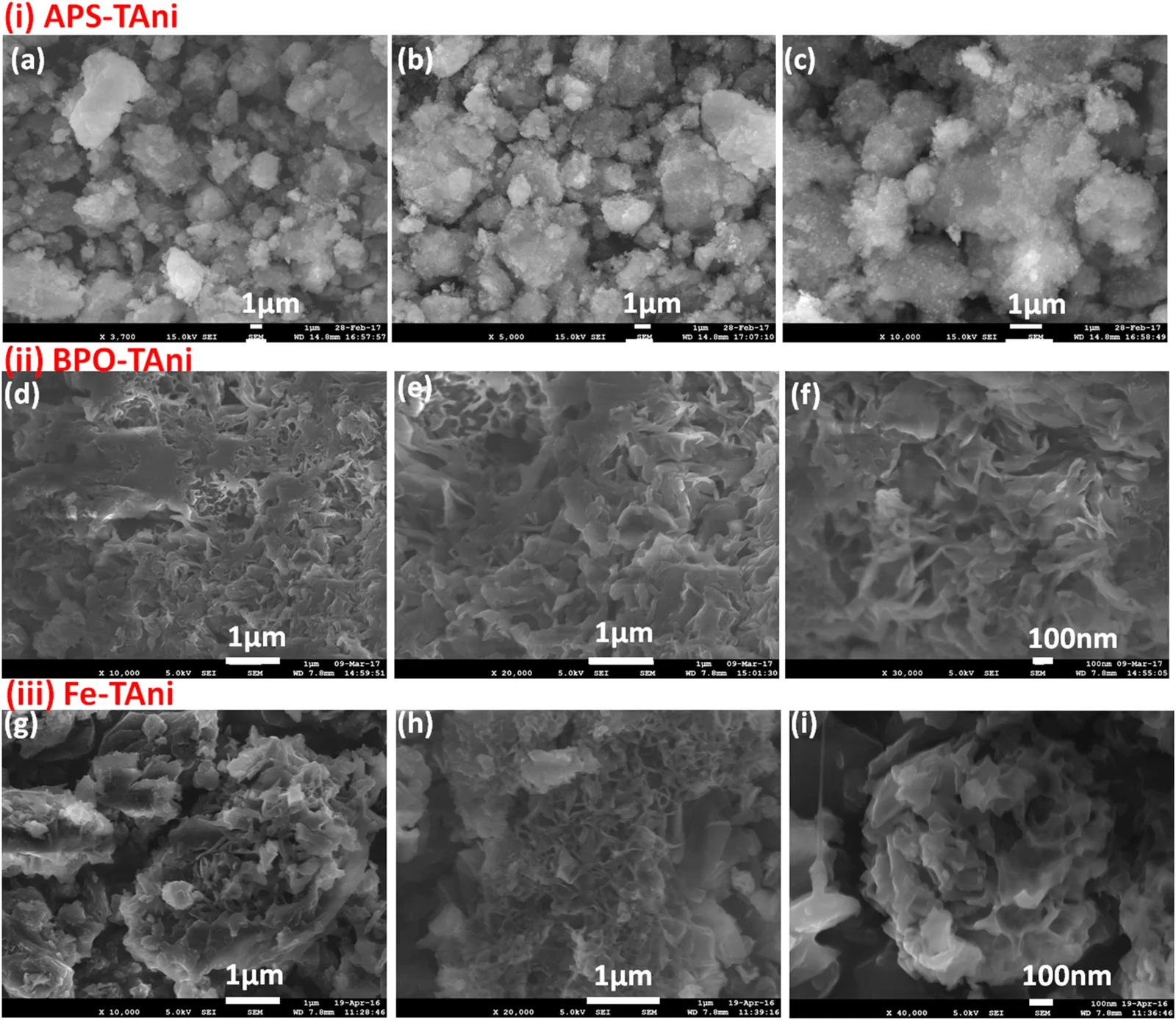
Field emission scanning electron micrograph (FE-SEM) images of emeraldine-base (i) APS-TAni, (ii) BPO-TAni and (iii) Fe-TAni at various magnifications.

**Fig. 19:**
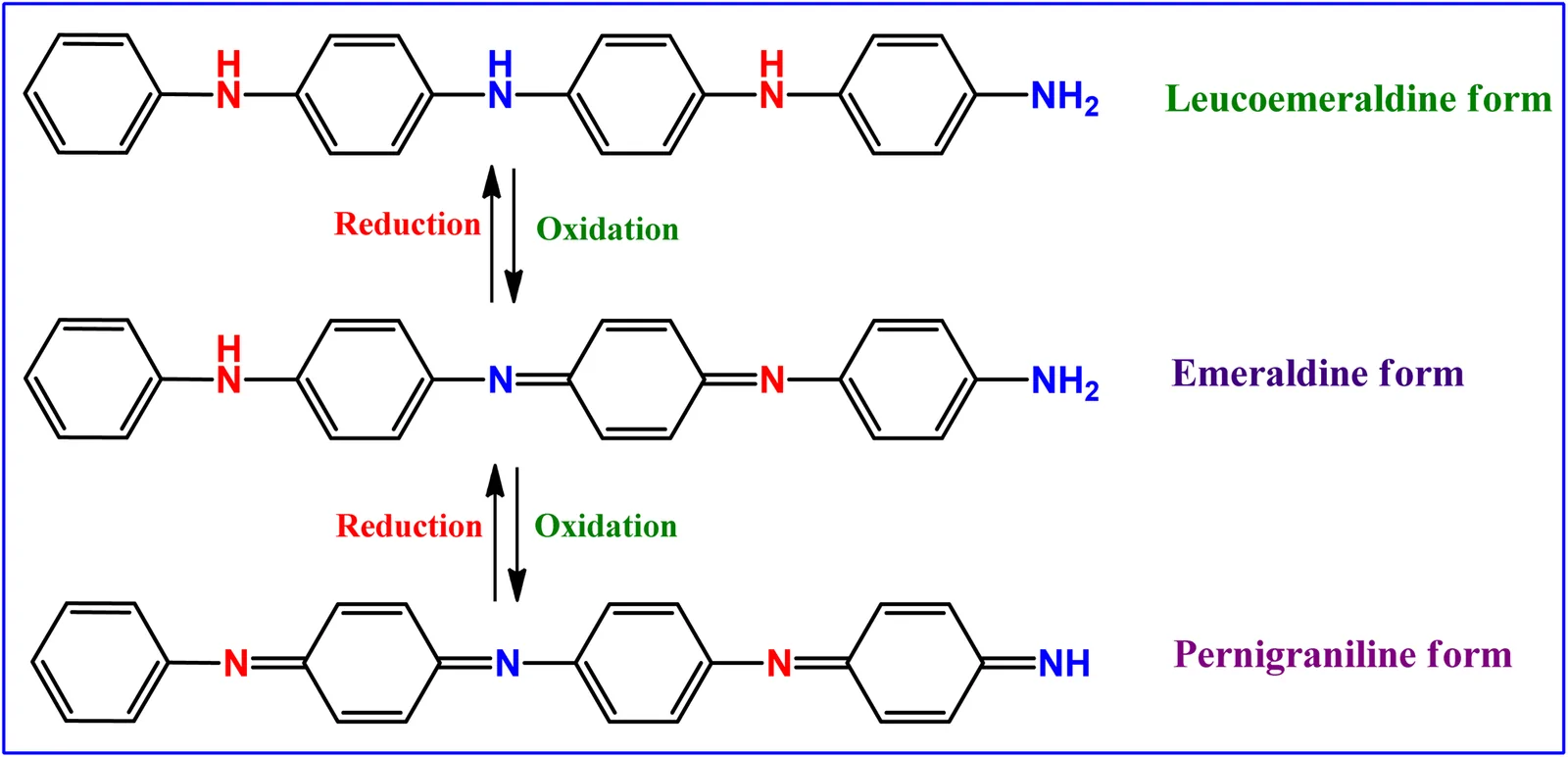
Molecular structures of TAni at various oxidation states.

### 4.5 Electrochemical studies

The redox behavior of the synthesized tetraaniline (TAni) samples was investigated using cyclic voltammetry (CV), a powerful technique for probing the electrochemical characteristics of conjugated oligomers and conducting polymers.^83^ Measurements were carried out in a three-electrode configuration using a platinum disc as the working electrode, a platinum wire as the counter electrode, and a saturated calomel electrode (SCE, Hg/Hg_2_Cl_2_) as the reference. The experiments were performed in DMF (20 mL) containing 1.0 M H_2_SO_4_ (5 mL) as a supporting electrolyte at a scan rate of 100 mV s^−1^ under a nitrogen atmosphere to prevent oxidative interference. The voltammograms of CAN-TAni, CC-TAni, APS-TAni, BPO-TAni, and Fe-TAni are presented in Fig. 20 and 21, and the electrochemical parameters are summarized in Table 7. It should be noted that no specific *iR*-drop compensation or liquid-junction-potential correction was applied during the CV measurements. The measurements were performed under identical experimental conditions for all TAni samples to enable consistent comparison of their electrochemical responses. Although the three redox couples remained well-defined and quasi-reversible during the recorded CV measurements, extended potential-cycling experiments were not systematically performed. Therefore, their long-term electrochemical stability was not evaluated in the present study.

**Fig. 20:**
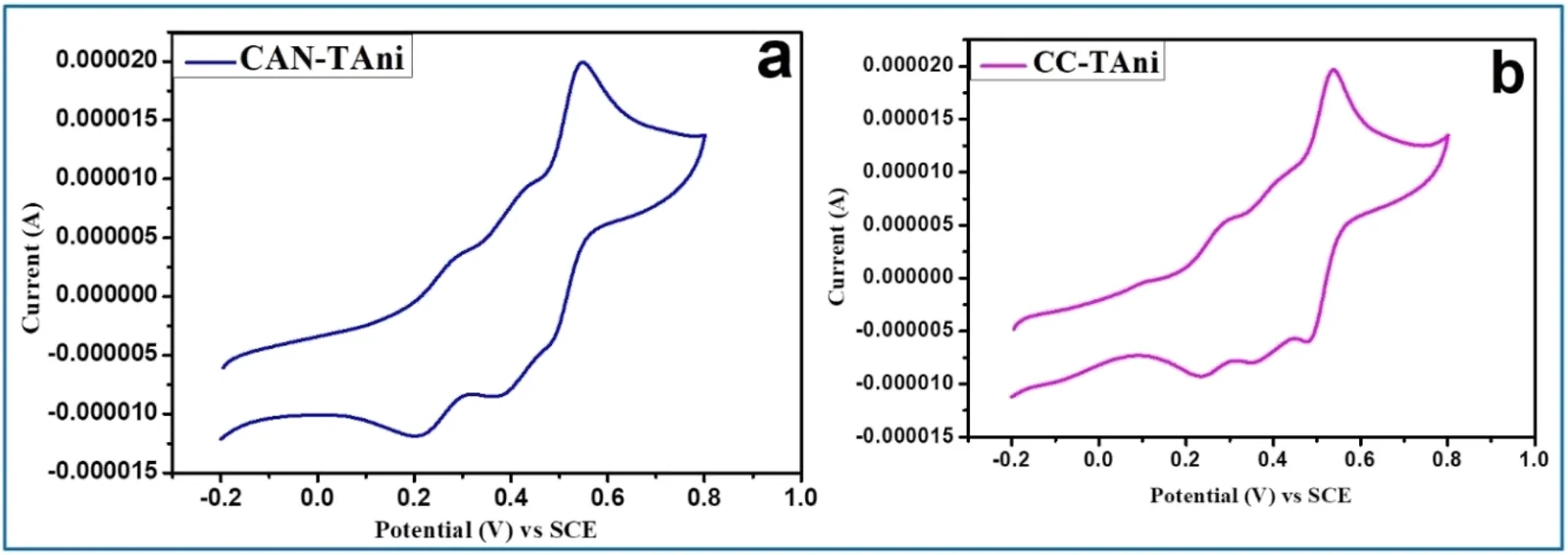
Cyclic voltammetry curves of (a) CAN-Tani and (b) CC-Tani in DMF (20 mL) and 1 M H_2_SO_4_ (5 mL) aqueous solution *vs.* SCE with a scan rate of 100 mV s^−1^.

**Fig. 21:**
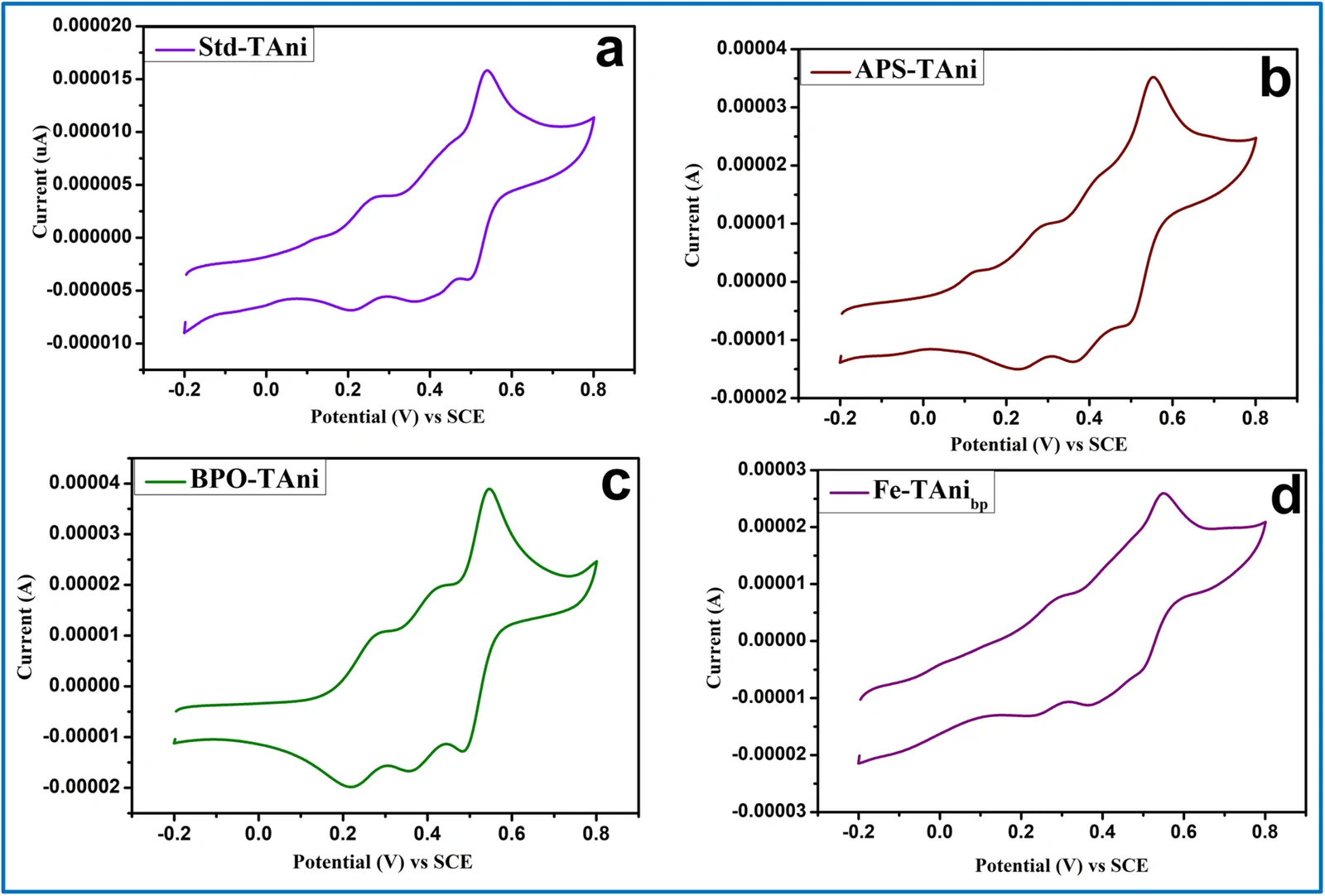
Cyclic voltammetry curves of (a) Std-TAni, (b) APS-TAni, (c) BPO-TAni and (d) Fe-TAni_bp_ in DMF (20 mL) and 1 M H_2_SO_4_ (5 mL) aqueous solution *vs.* SCE with a scan rate of 100 mV s^−1^.

**Table 7:** Cyclic voltammetry data of CS-TAni, CAN-TAni, CC-TAni, APS-TAni, BPO-TAni and Fe-TAni_bp_ in DMF (20 mL) and 1 M H_2_SO_4_ (5 mL) solution with a scan rate of 100 mV s^−1^

Polymer^a^	Oxidation peaks (V)			Reduction peaks (V)		
	Peak 1	Peak 2	Peak 3	Peak 1	Peak 2	Peak 3
Std-TAni	0.28	0.43	0.54	0.22	0.37	0.50
CAN-TAni	0.29	0.42	0.54	0.24	0.37	0.48
CC-TAni	0.29	0.43	0.55	0.22	0.38	0.50
APS-TAni	0.29	0.43	0.55	0.22	0.36	0.50
BPO-TAni	0.29	0.43	0.55	0.22	0.37	0.50
Fe-TAni_bp_	0.29	0.42	0.55	0.23	0.36	0.50

a CS – chemical synthesis; CAN – ceric ammonium nitrate; CC – copper(ii) chloride dihydrate; APS – ammonium peroxodisulfate; BPO – benzoyl peroxide; Fe – ferric chloride; bp – biphase.

All samples exhibited three well-defined and quasi-reversible redox couples within the investigated potential window, characteristic of oligoaniline systems (Fig. 20 and 21). The first anodic peak at 0.29 V corresponds to the oxidation from the LEB form to the intermediate EB (1) form containing one quinoid ring, and the second peak at 0.42 V is associated with further oxidation of the LEB form to EB (2)-TAni consisting of a quinoid ring. The third oxidation peak at 0.55 V is assigned to the transition from the emeraldine state to the fully oxidized pernigraniline base (PNB) form.^24,84^ The CV data are summarized in Table 7. This behavior is the same as that of chemically prepared TAni (Std-TAni) in the literature,^55^ and suggests that the CAN-TAni, CC-TAni, APS-TAni, BPO-TAni and Fe-TAni_bp_ are not different from chemically synthesized tetraaniline (Std-TAni), which is in EB form, and are equivalent in chemical structure. The leucoemeraldine structure was converted first into the emeraldine and then the pernigraniline structure, as observed in polyaniline. Overall, the CV results demonstrate that the synthesized tetraanilines exhibit reversible and well-defined redox activity analogous to that of polyaniline. This behavior confirms preservation of the conjugated amine–imine structure and highlights the suitability of these tetramers as model compounds for understanding redox processes in conducting polymers, as well as for potential applications in electrochemical devices and sensing platforms.

## 5. Conclusion

This study demonstrated the systematic synthesis of tetraaniline (TAni) through chemical oxidative coupling using a series of oxidizing agents, including ceric ammonium nitrate, copper(ii) chloride, benzoyl peroxide, ammonium persulfate, and iron(iii) chloride. The biphasic interfacial approach employed for Fe-TAni involving chloroform and water provided an effective route for controlled oligomer formation at the liquid–liquid interface. Although nanofibrous architectures analogous to polyaniline were anticipated, the reactions predominantly yielded nanostructured porous morphologies, highlighting the sensitivity of supramolecular organization to synthetic conditions. The structure of the electroactive emeraldine base form of the obtained tetramers was characterized using FT-IR, ^1^H NMR spectroscopy, ESI-mass spectrometry, elemental analysis and UV-Vis spectroscopy. The spectroscopic results confirmed that all oxidizing systems produced the same fundamental emeraldine-base tetraaniline framework. However, the comparative study demonstrated that the choice of oxidant influences the physicochemical and functional characteristics of TAni, including morphology, thermal behavior, electrical conductivity, and electrochemical performance. Thus, the scientific significance of the alternative oxidants lies in their ability to modify material properties while retaining the desired tetraaniline backbone, providing opportunities to select an appropriate oxidizing system according to the targeted application. Analysis of thermal properties and morphology was carried out using TGA, DSC, and FE-SEM. The electrochemical properties of all the tetramers were studied using cyclic voltammetry. The successful formation of tetraaniline in the electroactive emeraldine base (EB) state was confirmed across all the preparation methods. The consistency of the spectroscopic signatures verified the preservation of the conjugated amine–imine backbone and the characteristic benzenoid–quinoid architecture. Upon protonation with 1 M HCl, the tetramers CAN-TAni, CC-TAni, APS-TAni, BPO-TAni, and Fe-TAni_bp_ all exhibited a significant enhancement in electrical conductivity, better than the 3.0 × 10^−3^ S cm^−1^ shown by chemically prepared Std. TAni in the literature. The improved performance is attributed to favorable nanostructured and porous morphologies that facilitate dopant diffusion and charge delocalization. The chemically synthesized undoped/emeraldine base form tetramers possess good thermal stability under nitrogen, with decomposition initiating above ∼240–250 °C and proceeding through a two-step degradation pathway. The obtained emeraldine-base tetramers also showed a well-defined glass transition temperature (*T*_g_) and melting point (*T*_M_) around 89.78–95.78 °C and 124–168 °C, indicating uniform phase behavior and a degree of structural ordering. The surface morphology of the tetramers showed flake-like, spherulitic, and porous nanostructures depending on the oxidizing system, underscoring the influence of the synthetic route on micro- and nanoscale organization. The most important property is the electrochemical activity of the tetramers (in DMF solution) doped with aqueous sulfuric acid, which was investigated and compared with that of Std-TAni. All the tetramers exhibited three redox peaks, revealing that these tetramers have three oxidation and reduction peaks due to the transition of electrons through the LEB → EB1 → EB2 → PN states, closely resembling the redox behavior of polyaniline. Overall, the present work establishes that controlled chemical oxidation routes can produce structurally well-defined, thermally stable, and electrochemically active tetraaniline with enhanced electrical properties. The ability to tailor morphology and conductivity through synthetic strategies provides valuable insight for the rational design of oligoaniline-based materials for applications in organic electronics, sensing, and redox-active devices.

## Author contributions

All authors contributed to the writing of the manuscript. All authors approved the final version of the manuscript.

## Conflicts of interest

The authors declare no competing financial interest.

## Data Availability

The data supporting the findings of this study are available within the article. The datasets generated and analysed during this work include detailed experimental procedures, synthetic protocols, purification methods, and characterization data for emeraldine-base tetraaniline (TAni) synthesized using different oxidizing agents, namely ceric ammonium nitrate, copper(ii) chloride, ammonium persulfate, benzoyl peroxide, and iron(iii) chloride. Structural characterization data include FT-IR, ^1^H NMR, ESI-MS, CH elemental analysis, and UV-Vis spectroscopy, which confirm the successful synthesis and molecular structure of the prepared oligomers. Thermal and morphological characterization data comprise thermogravimetric analysis (TGA), differential scanning calorimetry (DSC), and field-emission scanning electron microscopy (FE-SEM). Electrical conductivity measurements of the HCl-doped samples and electrochemical characterization obtained by cyclic voltammetry are also included and provide the basis for the conclusions regarding the conductivity and redox behavior of the synthesized materials. All processed data used to generate the figures, tables, and conclusions presented in this manuscript are included in the article or the SI. Additional raw experimental data, spectra, and supporting records are available from the corresponding author upon reasonable request. No restrictions have been placed on access to these data. The authors have not deposited the datasets in a public repository because all data necessary to validate the findings and reproduce the reported results are provided in the manuscript.
